# Human CST Stimulates Base Excision Repair to Prevent the Accumulation of Oxidative DNA Damage

**DOI:** 10.1016/j.jmb.2024.168672

**Published:** 2024-06-20

**Authors:** Brandon C. Wysong, P. Logan Schuck, Madhumita Sridharan, Sophie Carrison, Yuichihiro Murakami, Lata Balakrishnan, Jason A. Stewart

**Affiliations:** 1 Department of Biology, School of Science, Indiana University, Indianapolis, IN, USA; 2 Department of Biological Sciences, University of South Carolina, Columbia, USA; 3 Department of Biology, Western Kentucky University, Bowling Green, KY, USA

**Keywords:** CST complex, oxidative damage, base excision repair (BER), DNA repair

## Abstract

CTC1-STN1-TEN1 (CST) is a single-stranded DNA binding protein vital for telomere length maintenance with additional genome-wide roles in DNA replication and repair. While CST was previously shown to function in double-strand break repair and promote replication restart, it is currently unclear whether it has specialized roles in other DNA repair pathways. Proper and efficient repair of DNA is critical to protecting genome integrity. Telomeres and other G-rich regions are strongly predisposed to oxidative DNA damage in the form of 8-oxoguanines, which are typically repaired by the base-excision repair (BER) pathway. Moreover, recent studies suggest that CST functions in the repair of oxidative DNA lesions. Therefore, we tested whether CST interacts with and regulates BER protein activity. Here, we show that CST robustly stimulates proteins involved in BER, including OGG1, Pol β, APE1, and LIGI, on both telomeric and non-telomeric DNA substrates. Biochemical reconstitution of the pathway indicates that CST stimulates BER. Finally, knockout of STN1 or CTC1 leads to increased levels of 8-oxoguanine, suggesting defective BER in the absence of CST. Combined, our results define an undiscovered function of CST in BER, where it acts as a stimulatory factor to promote efficient genome-wide oxidative repair.

## Introduction

The generation of reactive oxygen species (ROS) is an unintended consequence of cellular metabolism, which triggers progressive oxidative damage to the genome and, ultimately cell death. Efficient repair of the damage, generated by ROS, is crucial to prevent mutagenesis and maintain genome stability. Guanine bases are particularly prone to oxidative damage due to having a low redox potential. Therefore, G-rich regions, such as telomeres and CpG islands, are especially prone to oxidative damage in the form of 8-dihydro-2′-deoxyguanine (8-oxoG). 8-oxoG is the most common DNA lesion, and estimates are that 1.5 × 10^5^ spontaneous 8-oxoGs are formed in the genome each day due to normal metabolic processes.^1^

Exposure to harmful ultraviolet light, ionizing radiation, inflammation, and environmental pollutants, such as chemical carcinogens and tobacco smoke, can significantly compound the volume of ROS and, thus, 8-oxoGs produced in the cell.^2^ When 8-oxoGs are not repaired, they are highly mutagenic and can lead to the development of cancer and other aging disorders. Furthermore, high amounts of oxidative stress can accelerate the rate of telomere shortening, which can contribute to telomere biology disorders.^3^ High amounts of oxidative damage at telomeres are also linked to the onset of diabetes^4^ and cancer.^5^

Consequently, it is imperative that oxidative lesions are correctly repaired. The most significant pathway used to repair single base lesions is base excision repair (BER).^6^ BER is initiated through the action of DNA glycosylases, which locate and remove oxidized, deaminated, or other inappropriate bases.^7^ Amongst the various oxidized lesions, generation of 8-oxo-guanine (8-oxo-G) is the most frequently occurring one.^8,9^ This lesion is recognized by 8-oxo-guanine glycosylase (OGG1), that binds the lesion and cleaves the N-glycosidic bond, releasing the damaged base and generating an abasic site. It can further catalyze the cleavage of the phosphodiester bond generating a 5′phosphate (5′P) and 3′ α,β-polyunsaturated aldehyde (3′PUA) end, which is processed in the next step.^10^ Abasic sites generated by DNA glycosylases is cleaved by AP endonuclease 1 (APE1) to form a single-strand nick, containing a 3′hydroxyl (3′OH) and 5deoxyribose phosphate end (5′dRP), that can then be repaired via DNA polymerase β (Pol β) through one of two pathways: short patch BER [SP-BER] (in which a single nucleotide gets replaced)^11–13^ or long patch BER [LP-BER] (in which approximately 2–13 nucleotides are replaced).^13–16^ Typically, the LP-BER pathway is employed when the 5′dRP moiety is either oxidized or reduced, thereby inhibiting the lyase activity of Pol β. The polymerase then performs strand displacement synthesis to create a displaced flap which is cleaved by flap endonuclease (FEN1) to remove the modified 5′dRP moiety.

While the basic mechanisms of BER have been characterized, whether additional accessory factors are required in response to different DNA structures/sequences and chromatin environments is still not well understood. For example, the shelterin complex, a six-membered protein complex essential for telomere protection, has been implicated in stimulating the BER pathway *via* several mechanisms including the stimulation of Pol β.^17,18^ Here, we tested whether another telomere-associated complex, CTC1-STN1-TEN1 (CST), also stimulates BER. CST is a single-stranded DNA (ssDNA) binding protein, which shares structural similarity to Replication Protein A (RPA).^19–21^ It primarily functions in the replication and maintenance of telomeres, where it coordinates telomere duplex replication and telomerase-dependent telomere elongation.^20^ CST also functions in various aspects of DNA replication and repair, including origin licensing, the rescue of stalled replication, and double-strand break repair.^21–23^ Mutations in CST are associated with the telomere biology disorders dyskeratosis congenita, Coats plus, and idiopathic pulmonary fibrosis as well as increased risk of various cancers.^21^

Previous work demonstrated that CST has preference for binding to G-rich DNA, such as telomeres and CpG islands, and can resolve DNA secondary structures called G-quadruplexes (G4s).^24,25^ Similar to RPA, CST can recruit and/or enhance the enzymatic activity of several proteins to promote various DNA maintenance pathways, including telomere replication, DNA replication restart, and double-strand break repair. A well-known function of CST is its ability to interact with and stimulate the activity of DNA polymerase α-primase (Pol α).^26–32^ Recent findings from two independent groups identified links between defective oxidative DNA repair and CST deficiency, suggesting a potential function of CST in the repair of oxidative DNA damage.^33,34^ Work by Nguyen et al. demonstrated that conditional deletion of STN1 increased the levels of oxidative DNA damage and resulted in increased tumor incidence and burden in a colorectal mouse model. Furthermore, another study by Hara et al., investigating the effects of H_2_O_2_ treatment in STN1 depleted cells, showed that cells lacking CST are particularly sensitive to H_2_O_2_-induced DNA damage, leading to defective RAD51 foci formation and S-phase progression. These findings suggest CST may participate in the repair of oxidative DNA damage, potentially through the modulation of BER. However, how CST functions in this process remains poorly defined. Therefore, we sought to directly test the extent to which CST promotes BER *in vitro* as well as determine whether CST associates with BER factors in cells. Since CST stimulates Pol α, we postulated that CST may also interact with and regulate the activity the BER polymerase Pol β.

Consistent with our hypothesis, we find that CST interacts with Pol β and stimulates its various activities *in vitro*. Furthermore, we tested the individual components of the BER pathway and found that the CST complex interacts with other BER components and stimulates multiple steps in the repair process on both telomere and non-telomere substrates. Biochemical reconstitution of the entire long patch BER pathway demonstrated that CST greatly stimulates BER *in vitro*. In line with these findings, deletion of STN1 or CTC1 increased genomic 8-oxo-Gs, indicating a defect in BER. Overall, our findings strongly support a role for CST in the repair of oxidative DNA damage through stimulation of BER.

## Results

### STN1 interacts with BER components

**Figure 1.**
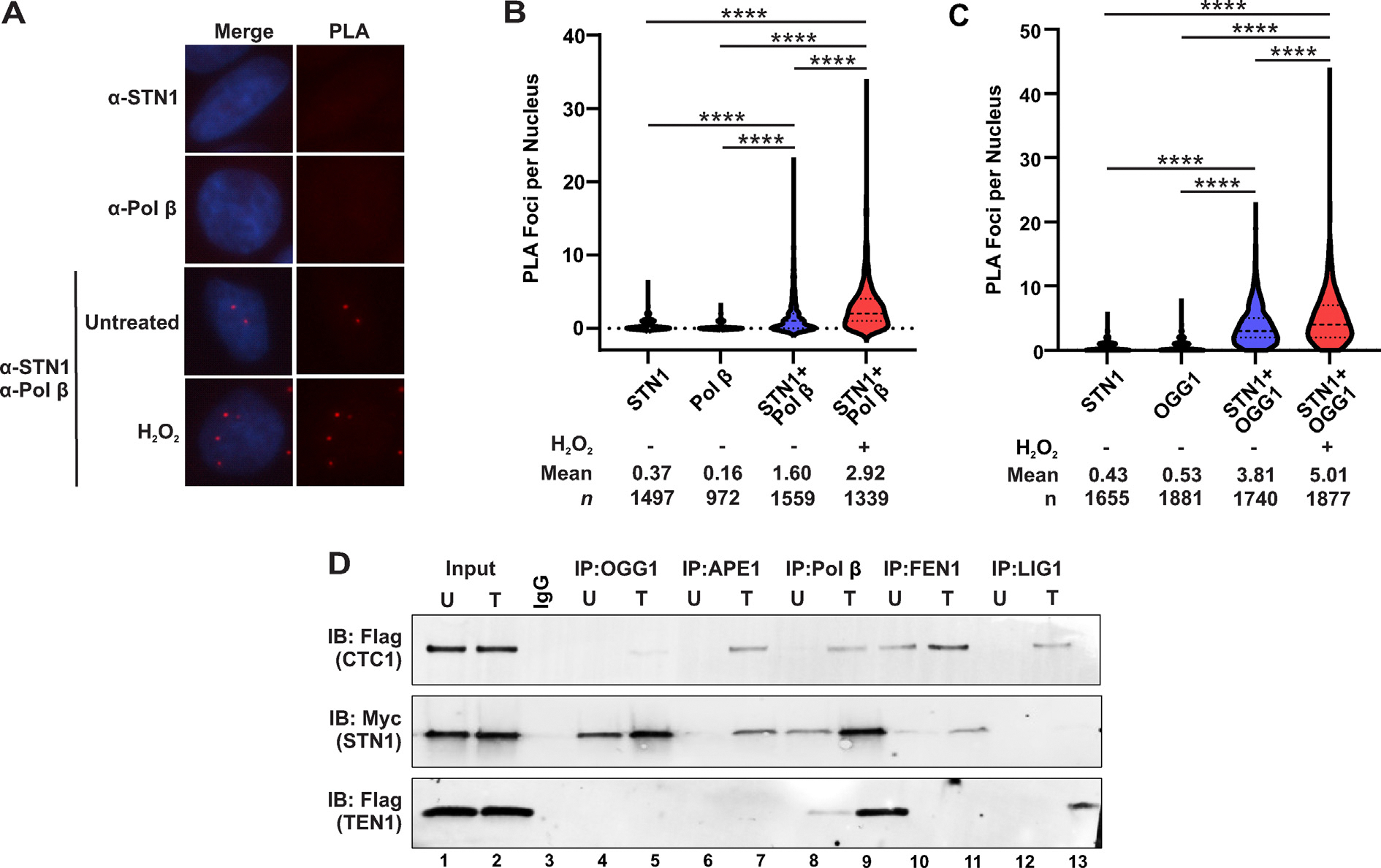
CST associates with BER proteins. (A) Representative images of proximity ligation assay (PLA) performed in HeLa cells with antibodies to STN1 or Pol β alone or in combination with and without H_2_O_2_ treatment. Red, PLA foci; blue, DAPI. (B) Violin plot of PLA foci per nucleus. Results are representative of three independent, biological experiments. Bold dashed line represents the median, and dashed lines represent the first and third quartiles. (C) Proximity ligation assay (PLA) performed in HeLa cells with antibodies to STN1, OGG1, or in combination (STN1 + OGG1). *n* = 3 independent, biological experiments. Bold dashed line: median, dashed lines: first and third quartiles. (D) HEK293T cell extracts containing individually overexpressed CST components (either FLAG-CST, Myc-STN1 or FLAG-TEN1) that were either untreated or treated with 200 μM of H_2_O_2_ were used to immunoprecipitate APE1, Pol β, FEN1 or LIGI and immunobloted with either anti-FLAG (for CTC1 and TEN1) or anti-Myc for STN1. Lysates were pre-treated with benzonase prior to coimmunoprecipitation. Proteins are identified in the Western blot analysis. (****p < 0.0001).

Pol β plays a crucial role in maintaining fidelity of the genome through its function in BER, where it synthesizes new bases to replace those that were damaged.^6^ Since CST is known to interact with and stimulate pol α, we performed proximity ligation assay (PLA)^35^ with antibodies to endogenous STN1 and Pol β. In comparison to single antibody controls, we observed an increase in the number of PLA foci with antibodies to STN1 and Pol β (Figure 1A, B). Next, we treated cells with H_2_O_2_ to determine whether oxidative stress increased the association between CST and Pol β. Treatment with H_2_O_2_ led to an approximate 2-fold increase in PLA foci, suggesting that their interaction is linked to oxidative stress and potentially BER (Figure 1B). Since the primary role of Pol β is in BER, we hypothesized that CST interacts or associates with other BER components, so we next tested whether OGG1 and STN1 interact by PLA and observed a strong interaction between OGG1 and STN1 (Figure 1C). PLA was then performed with antibodies to other factors that function in BER, including PARP1 and XRCC1. Like Pol β and OGG1, STN1 associated with PARP1 and XRCC1 (Figure S1A, B). As a control, we also performed PLA for Pol β-XRCC1, which showed a strong interaction as expected (Figure S1C).

To further validate the PLA data, we performed coimmunoprecipitation (Co-IP) experiments to explore whether individual components of the CST complex interacted with BER-associated proteins. Individual components of the CST complex (Flag-CTC1, Myc-STN1, or Flag-TEN1) were overexpressed in HEK293T cells for 24 h. Cells were then either left untreated or treated with 200 μM H_2_O_2_ for 2 h prior to performing immunoprecipitation assays. Since it was previously reported that CST plays a role in oxidative damage, we were interested in observing if interactions between CST components occurred in undamaged cells or if they only occurred in the presence of oxidative damage (H_2_O_2_ treatment). To ensure that interaction was not mediated by DNA, lyastes were treated with benzonase prior to Co-IP. Individual BER proteins (OGG1, APE1, Pol β, FEN1 and LigI) were immunoprecipitated and immunoblotted with either anti-Flag (to detect CTC1 or TEN1) or anti-Myc (to detect STN1) antibody (Figure 1D). Interestingly, CST subunits showed varied interaction with different BER proteins. STN1 showed a strong interaction with OGG1 in both the presence and absence of H_2_O_2_, whereas CTC1 showed an extremely weak interaction in the presence of H_2_O_2_ and TEN1 showed no detectable interaction (lanes 4–5, Figure 1D). CTC1 and STN1 interacted with APE1 in the presence of H_2_O_2_, but no interactions were observed with TEN1 (lanes 6–7, Figure 1D). All three subunits (CTC1, STN1 and TEN1) interacted with Pol β, with STN1 showing interactions even in the absence of oxidative damage (lanes 8–9, Figure 1D). CTC1 and STN1 interacted with FEN1 in the presence or absence of oxidative damage, albeit CTC1 showed greater interaction compared to STN1. TEN1 showed no interaction with FEN1 (lanes 10–11, Figure 1D). CTC1 and TEN1 components also showed interacted with LigI in the presence of oxidative damage, with TEN1 showing the highest binding efficiency and both CTC1 displaying a weak interaction (lanes 12–13, Figure 1D).

Together, these findings suggest that the CST components interact with various proteins in the BER pathway, strongly suggesting a potential function of CST in BER.

### CST enhances multiple steps in BER in vitro

To better understand how CST may promote BER, we sought to characterize how CST and individual CST components affected the different steps of BER through biochemical reconstitution, using purified proteins and DNA substrates that resemble the different intermediates of both SP- and LP-BER. We first confirmed that our purified CST was active by observing DNA binding on a ssDNA oligonucleotide (Figure S2). Consistent with previous studies,^36–38^ individual subunits showed weak to undetectable binding in comparison to the full CST complex (Figure S2B). Since our PLA results suggested that within the cell, some components on the CST complex were in a complex with BER proteins, we next tested to see if the recombinant proteins used in our biochemical assays interact with each other. For the binding assays, we incubated the CST complex with individual components of the BER pathway (OGG1, APE1, Pol β, FEN1 and LigI) in a 1:1 ratio for 2 h and subsequently incubated the binding reactions on Flag beads for 4 h to pulldown Flag-CTC1. Interactions between CST and the BER proteins were probed by assaying the input (I), flowthrough (F) and eluate (E) using an anti-His antibody (since all BER recombinant proteins are 6X-His tagged). Results showed that all BER proteins interacted with the CST complex (eluate lanes 3, 6, 9, 12, 15, Figure S3). However, we also observed bands in the flowthrough fractions (F) suggesting that not all BER proteins bound to the CST complex in a 1:1 ratio (Figure S3).

#### CST stimulates OGG1 cleavage.

**Figure 2.**
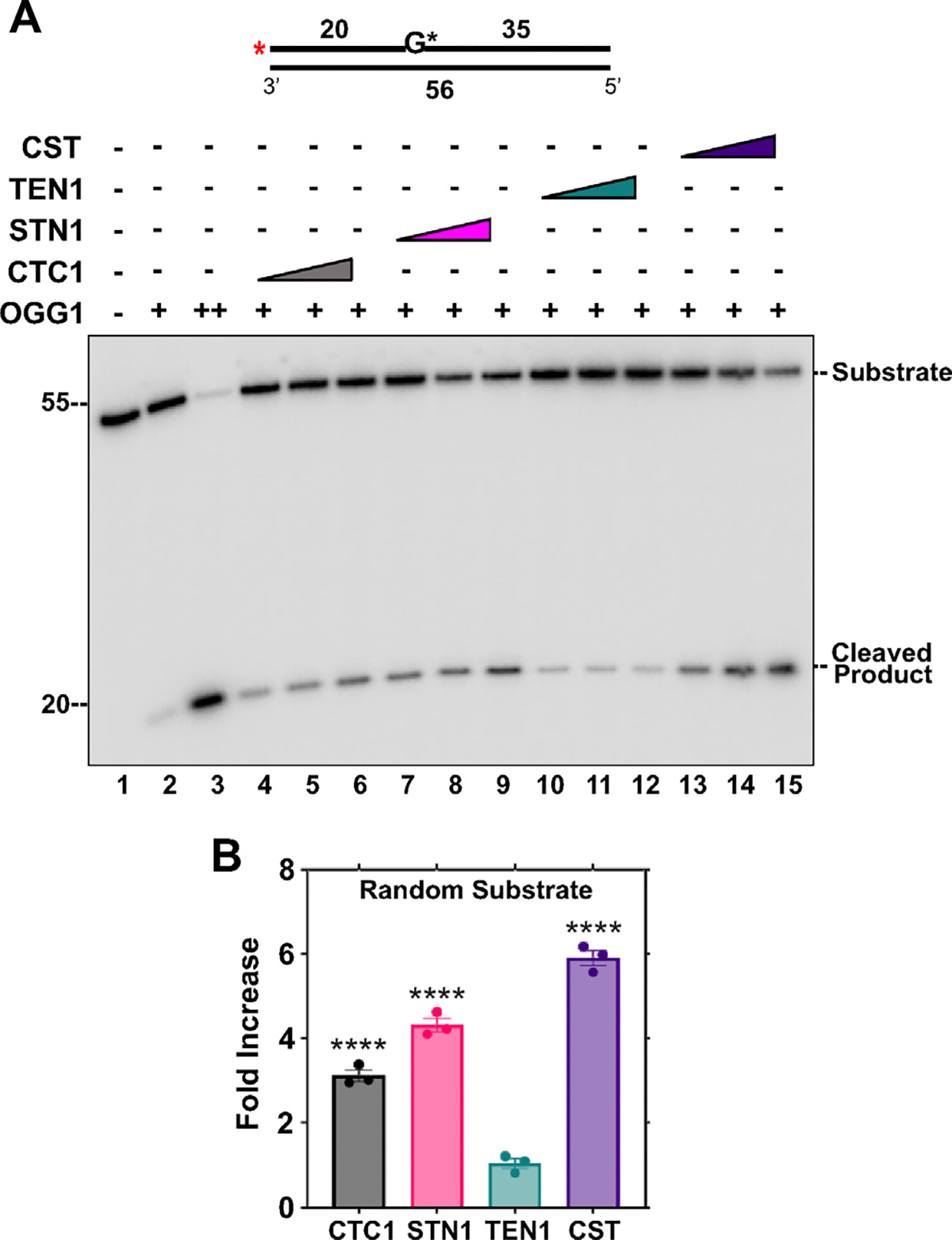
The CST complex and individual subunits stimulate OGG1 activity on 8-oxo-G containing substrate. (A) OGG1 (2 nM, lanes 2, 4–15; 50 nM, lane 3) lesion base removal and phosphodiester bone cleavage activity were measured on a random sequence DNA substrate (5 nM) containing an 8-oxo-guanine (8-oxo-G) residue in the presence of increasing concentrations of CTC1/STN1/TEN1 (250, 750, 1500 nM) or CST (5, 15, 60 nM) as denoted in the figure. The red asterisk shown on the substrate in the figure represents the location of the ^32^P radiolabel and a G* represents an 8-oxo-G residue. Gel shown is representative of at least three independent experiments. (B) Graph of the fold increase in OGG1 cleavage activity for the lanes containing the highest concentration of the CST complex and individual subunits. The random substrate (U1:T1) was generated by annealing primers in a 1:2 ratio. (****p < 0.0001).

OGG1 is a glycosylase that recognizes oxidized guanines and removes the lesion to create an abasic site. It can also cleave the phosphodiester backbone creating a nicked substrate. The impact of the presence of either individual components of CST or the entire complex on the activity of OGG1 on a lesion containing substrate was evaluated. Removal of the 8-oxo-G lesion and cleavage of the phosphodiester backbone were analyzed on a denaturing gel (Figure 2A) and fold change in activity calculated. Removal of lesion and cleavage activity of OGG1 was increased by 3.1-fold in the presence of CTC1, 4.3-fold in the presence of STN1 and 5.9-fold in the presence of the full CST complex (Figure 2B). The presence of TEN1 did not improve OGG1 activity, which was not surprising, since TEN1 did not seem to interact with OGG1 in the Co-IP assay, whereas STN1 showed strong and CTC1 weak interaction with OGG1 (Figure 1C).

#### CST stimulates APE1 cleavage.

**Figure 3.**
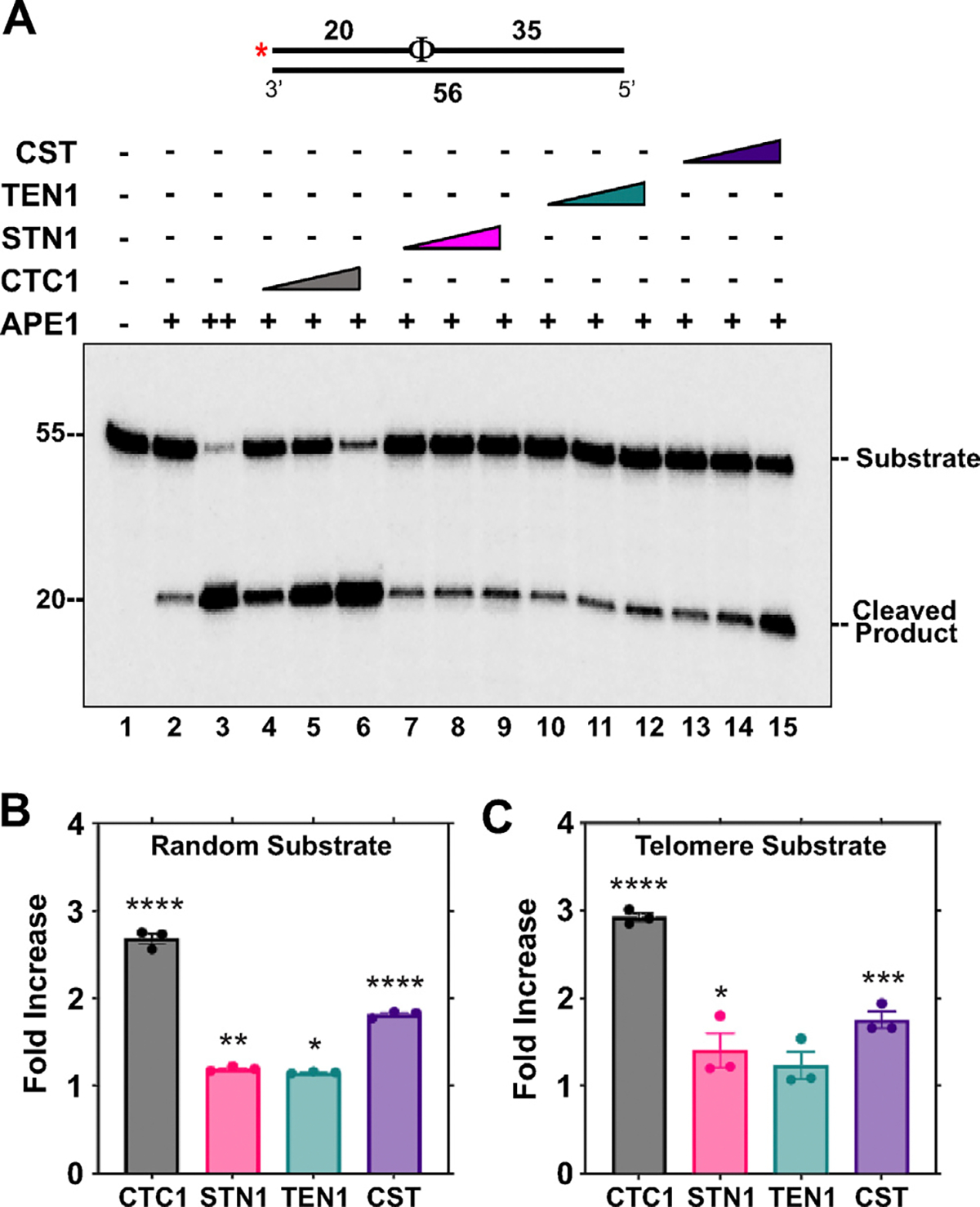
The CST complex and CTC1 enhance APE1 cleavage activity. (A) APE1 (0.075 nM, lanes 2, 4–15; 100 nM, lane 3) cleavage activity was measured on a random sequence DNA substrate (5 nM) containing a THF residue in the presence of increasing concentrations of CTC1/STN1/TEN1 (250, 750, 1500 nM) or CST (5, 15, 60 nM) as denoted in the figure. The red asterisk shown on the substrate in the figure represents the location of the ^32^P radiolabel and an Φ represents a THF residue. Gel shown is representative of at least three independent experiments. (B) Graph of the fold increase in APE1 cleavage activity for the lanes containing the highest concentration of the CST complex and individual subunits. (C) Graph of the fold increase in APE1 cleavage activity on a telomere substrate calculated as in (B). The error bars represent the standard error mean. The random substrate (U2:T1) and telomere substrate (U3:T2) was generated by annealing primers in a 1:2 ratio. (****p < 0.0001, ***p < 0.001, **p < 0.01, *p < 0.05).

During BER, APE1 cleaves the abasic site generated after a DNA glycosylase removes the oxidized or damaged base. APE1 is an indispensable player in BER, and its efficiency must be maximized for cells to cope with oxidative damage.^39,40^ Additionally, cleavage by APE1 is common between both SP- and LP-BER. To assess how CST affected APE1 activity, the individual subunits or the full CST complex was incubated with APE1 and the endonuclease activity of APE1 was tested on a DNA substrate containing a THF residue, which resembles an abasic site. On a random THF substrate (Figure 3A) APE1 endonuclease activity was modestly stimulated by the CST complex with a nearly 2.0-fold increase. CTC1 alone stimulated cleavage 2.7-fold and the addition of STN1 or TEN1 resulted in an approximately 1.2-fold increase (Figure 3B). Similar patterns of stimulation were observed on a telomeric THF substrate (Figure 3C).

#### CST stimulates Pol β activity.

**Figure 4.**
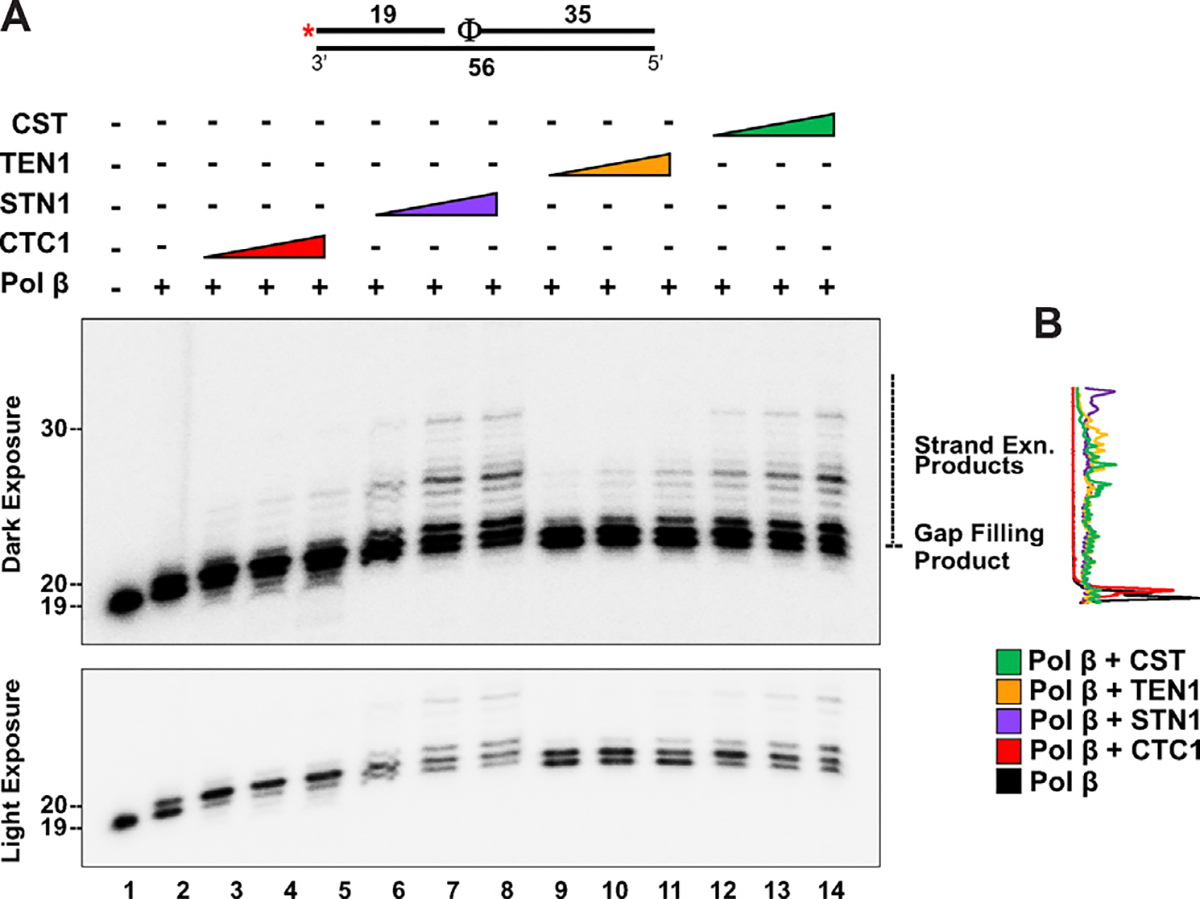
The CST complex and individual subunits stimulate DNA Pol β synthesis on a gap substrate containing an abasic site. Synthesis by DNA Pol β (2 nM) on a 1 nt gapped substrate (5 nM) containing an abasic site mimic (Φ denotes tetrahydrofuran [THF]) in the presence of increasing amounts of CTC1/STN1/TEN1 (100, 500, 1000 nM) or CST (5, 15, 30 nM) as denoted in the figure. The substrate was generated by annealing primers U4:T1: D1 in a 1:2:4 ratio. The red asterisk shown on the substrate represents the location of the ^32^P radiolabel. Gel shown is representative of at least three independent experiments. A darker exposure of the gel shows the strand displacement synthesis products. (B) The trace at the right of the gel depicts the signal intensity of the gap-filling and strand-displacement products in the lanes containing the highest concentration of CTC1(red trace)/STN1 (purple trace)/TEN1 (orange trace)/CST (green trace) [lanes 6, 10, 14, and 18] as compared to Pol β synthesis alone (black trace) [lane 2].

To study the impact of CST on Pol β, we first used a substrate containing a tetrahydrofuran (THF) residue next to a gap. The THF mimics an abasic (apurinic or apyrimidinic) site and the gap resembles a BER intermediate that has already been processed by APE1 (where the phosphodiester backbone has been cleaved at the abasic site).^41^ Using this substrate, we tested the ability of Pol β to fill in the gap either in the presence of increasing concentrations of individual CST components or the full CST complex. We observed that CTC1 and STN1 and the entire complex stimulated the gap-filling activity of Pol β (Figure 4A). Additionally, we observed increased strand displacement synthesis by Pol β in the presence of CTC1, STN1, and the entire CST complex. Synthesis traces are shown in Figure 4B.

Pol β has previously been shown to possess processive gap-filling activity on substrates containing gaps up to six nucleotides.^42,43^ Since we observed a stimulation in Pol β synthesis on a 1-nt gapped THF substrate, we next utilized a 6-nt gapped substrate to analyze the impact of CST on Pol β’s synthesis and strand displacement synthesis activities. Due to CST’s function at both telomeres and genome-wide, DNA synthesis and strand displacement were monitored on both a random sequence (Figure S4A, B) and a telomeric sequence (Figure S4C) containing substrate in the presence of increasing concentrations of the CST complex or individual subunits. Both CTC1 and STN1 were able to greatly improve the strand displacement activity of Pol β, with a calculated 3-fold increase in synthesis for both subunits. TEN1 and the CST complex were able to improve the strand-displacement activity of Pol β marginally, as evidenced by an increase in synthesis beyond the pause point, but not to the degree observed with CTC1 and STN1 (Figure S4A). Synthesis traces on random (Figure S4B) and telomere (Figure S4C) substrates display increased strand displacement synthesis in the presence of CST, irrespective of the substrate sequence. On the telomere substrate, both CTC1 (red trace, Figure S4C) and STN1 (purple trace, Figure S4C) showed increased full-length synthesis when compared to synthesis by Pol β alone (black trace, Figure S4C).

Unlike the replicative polymerases, Pol β does not synthesize over long stretches of a DNA template. However, since we observed varying levels of stimulation by the individual components as well as the entire CST complex, we next tested the impact of CST stimulation on the gap-filling function of Pol β. Similar to its stimulation of Pol α synthesis,^26,29,30^ CST stimulated Pol β synthesis on a random sequence containing 66 nt gapped DNA substrate. Incubation with CTC1 (lanes 4,5,6, Figure S4D) or STN1 (lanes 8,9,10, Figure SD) alone even resulted in the robust accumulation of full-length product, which was not achieved using Pol β alone. Both CTC1 and STN1 were shown to stimulate Pol β DNA synthesis by ~7-fold, whereas TEN1 (lanes 12, 13, 14, Figure S4D) or the entire CST complex (lanes 16, 17, 18, Figure S4D) resulted in an ~3-fold increase in synthesis. This increase was both observable and quantifiable, as shown in the intensity traces for both the random (Figure S4E) and telomere (Figure S4F) substrates.

#### CST stimulates Pol β lyase activity.

In addition to DNA synthesis *via* its polymerization domain, Pol β also possesses a lyase domain, which removes the dRP residue that persists following APE1 cleavage of an abasic site. Previous studies suggested the dRP lyase activity of Pol β occurred after gap-filling synthesis by the polymerase.^11,44,45^ A recent study has proposed an interlocked mechanism wherein the polymerase and dRP lyase function in tandem.^46^ Irrespective of the sequence of events, without lyase activity, SP-BER cannot be completed. Therefore, we next examined the impact CST has on the lyase activity of Pol β.

**Figure 5.**
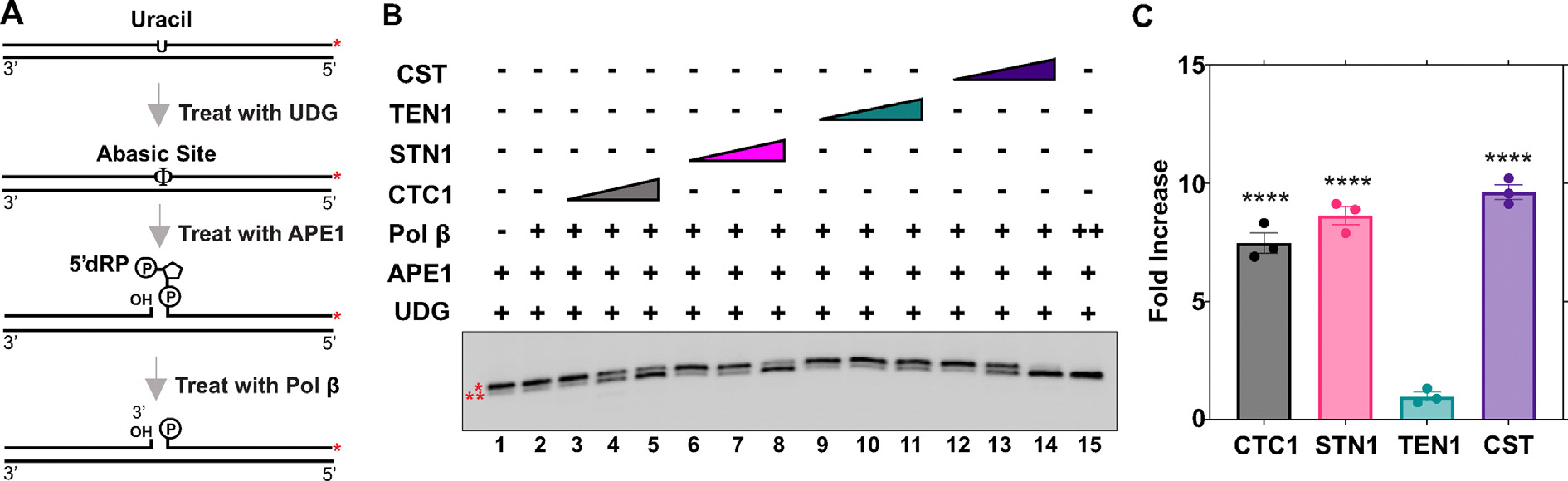
The CST complex, CTC1, and STN1 subunits stimulate dRP lyase activity of Pol β. (A) Substrate (5 nM) was pre-treated with 1 U of UDG before the addition of 100 nM APE1 to generate the dRP substrate, as shown in the schematic and described in “Materials and Methods” (B) The lyase activity of Pol β (0.25 nM, lanes 1–13; 5 nM, lane 14) was observed in the presence of increasing concentrations of CTC1/STN1/TEN1 (250, 750, 1500 nM) or CST (7.5, 15, 45 nM) as denoted in the figure. Gel shown is representative of at least three replicate experiments. (C) Graph of the fold increase in lyase activity for the lanes containing the highest concentration of CST proteins utilized. The error bars represent the standard error mean. * denotes the dRP lyase substrate; ** denotes the dRP lyase product. The substrate was generated by annealing primers U7:T1 in a 1:2 ratio. (****p < 0.0001).

To study the dRP lyase activity, we utilized a 55 nt duplex DNA substrate that contained an uracil residue. The uracil containing DNA was 3′ end labeled with [α-^32^P]dCTP and then treated with Uracil DNA Glycosylase (UDG) to generate an abasic site. APE1 cleavage of this abasic site forms a 5′-dRP group which can be excised via the lyase activity of Pol β. Because this Pol β-incised product does not contain the sugar phosphate residue, it will migrate slightly faster when separated by gel electrophoresis. A schematic of the methodology used to generate the substrate and perform the assay is outlined in Figure 5A.

CST or individual subunits were incubated with the lyase substrate. Lyase activity was then assessed by denaturing polyacrylamide gel electrophoresis. The results demonstrated that the lyase activity of Pol β is improved in the presence of the full complex and individual subunits (Figure 5B, C). There was an ~7.5-fold increase in Pol β lyase activity with CTC1, 8-fold by STN1, 1.5-fold with TEN1, and an ~10-fold increase with the full complex. These results provide further evidence that CST interacts with Pol β and mechanistically improves its catalytic functions during BER.

#### Individual subunits but not the CST complex stimulate FEN1.

**Figure 6.**
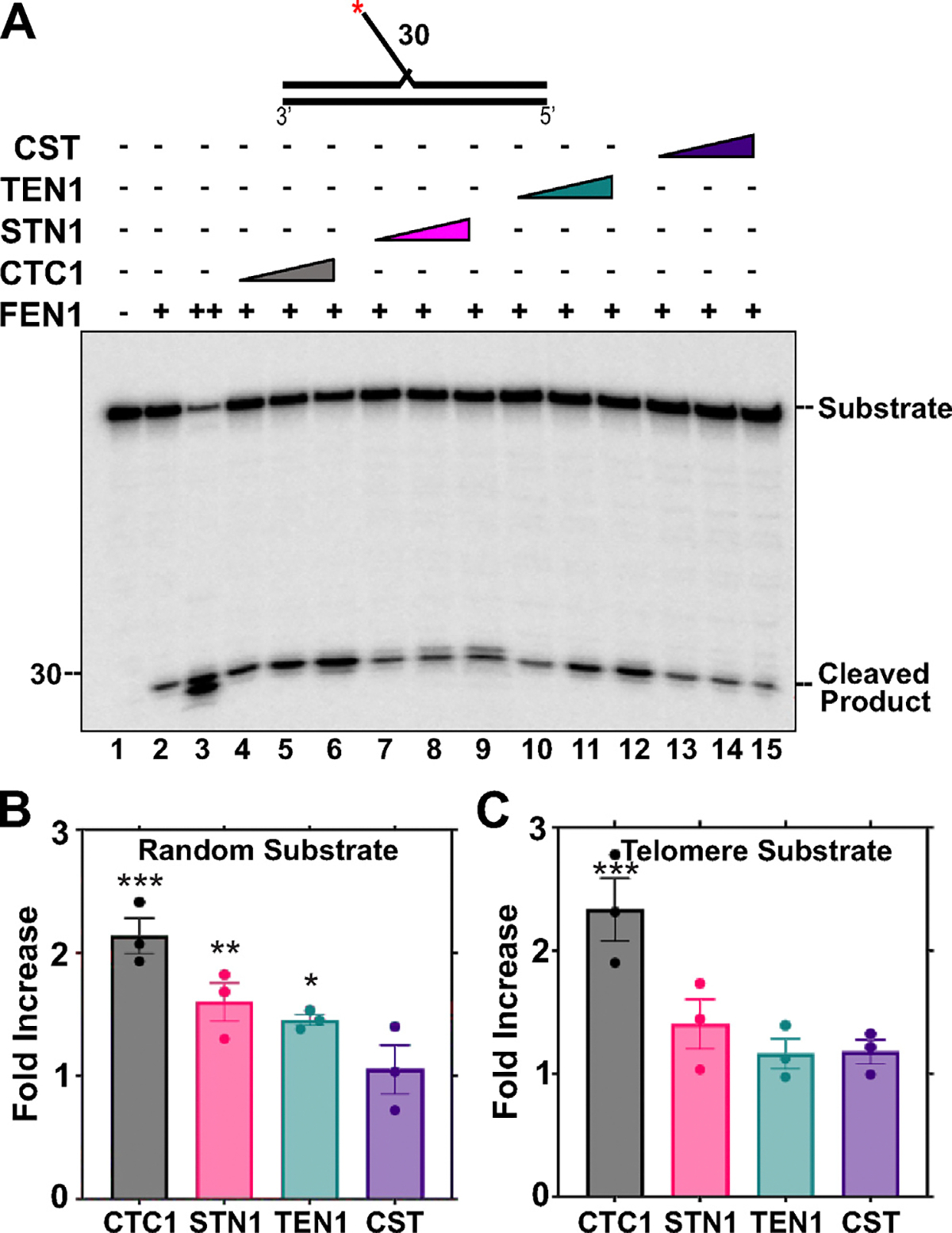
FEN1 cleavage activity is stimulated by CTC1 but not the full CST complex. FEN1 (0.025 nM, lanes 2, 4–15; 10 nM, lane 2) cleavage activity was measured on a random sequence 5′ DNA flap DNA substrate (5 nM) in the presence of increasing concentrations of CTC1/STN1/TEN1 (250, 750, 1500 nM) or CST (5, 15, 60 nM) as denoted in the figure. The red asterisk shown on the substrate in the figure represents the location of the ^32^P radiolabel. Gel shown is representative of at least three independent experiments. (B) Graph of the fold increase in FEN1 cleavage activity for each of the lanes containing the highest concentration of CST proteins utilized. (C) Shows the fold increase in FEN1 cleavage activity on a telomere substrate calculated as in (B). The error bars represent the standard error mean. The random substrate (U8:T5:D4) and telomere substrate (U9:T6:D5) was generated by annealing primers in a 4:2:1 ratio. (***p < 0.001, **p < 0.01, *p < 0.05).

Flap endonuclease 1 (FEN1) also plays an important role during LP-BER by excising the 5′ DNA flaps that are formed.^47^ Without functional or optimal FEN1, gap-filling stalls and the efficiency of LP-BER is significantly diminished.^48^ To test whether CST regulates FEN1 nuclease activity, CST or individual subunits and FEN1 were incubated with a DNA substrate with a 35 nt 5′ DNA flap. FEN1 cleavage was then measured (Figure 6A). A 2.1-fold stimulation in FEN1 activity was observed in the presence of CTC1 and ~1.5-fold stimulation measured with STN1 or TEN1 (Figure 6B). However, minimal effect was observed in the presence of the full CST complex. Similar results were observed with a telomere-sequence containing substrate, except that TEN1 showed minimal stimulation similar to the full complex (Figure 6C).

#### CST promotes ligase activity.

**Figure 7.**
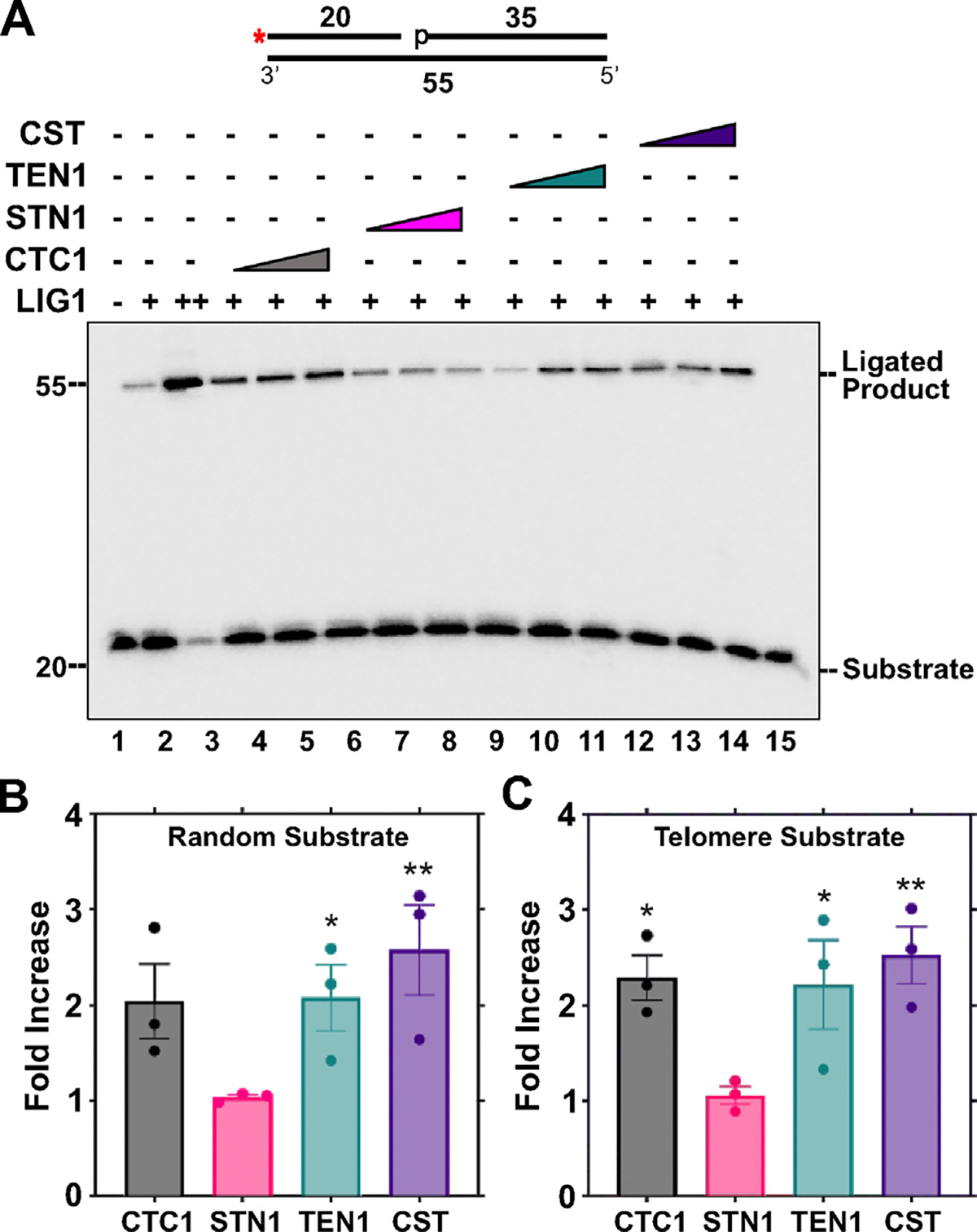
The CST complex, CTC1 and TEN1 stimulate ligation efficiency of LIGI. (A) LIGI (0.05 nM, lanes 2, 4–15; 100 nM, lane 3) ligation activity was measured on a nicked DNA substrate (5 nM) in the presence of increasing concentrations of CTC1/STN1/TEN1 (250, 750, 1500 nM) or CST (5, 15, 45 nM) as denoted. The red asterisk shown on the substrate in the figure represents the location of the ^32^P radiolabel. Gel shown is representative of at least three independent experiments. (B) Graph of the fold increase in LIGI ligation activity for the lanes containing the highest concentration of CST proteins utilized. (C) Shows the fold increase in LIGI ligation activity on a telomere substrate calculated as in (B). The error bars represent the standard error mean. The random substrate (U10:T1:D6) and telomere substrate (U11:T4:D7) was generated by annealing primers in a 1:2:4 ratio. (**p < 0.01, *p < 0.05).

LIGI is responsible for completing the final step of both SP-BER and LP-BER by sealing the nick between the DNA stands following Pol β gap filling.^49^ The ligation efficiency of LIGI was assessed in the presence of CST or individual subunits on a nicked DNA substrate (Figure 7A). CST, CTC1, or TEN1 increased the ligation activity of LIGI by ~2-fold, while STN1 showed no effect (Figure 7B). Similar results were observed with a nicked telomere-sequence containing substrate (Figure 7C). These results demonstrate that CST improves the ligation activity of LIGI on nicked DNA.

### CST enhances long patch BER

Based on the ability of CST and individual components to stimulate multiple steps of BER, we sought to reconstitute the entire LP-BER pathway *in vitro* and characterize the impact that CST and individual subunits have on the overall repair efficiency. LP-BER is employed by the cell when the dRP lyase activity of Pol β is diminished or inhibited.^50^ In this pathway, Pol β displaces the oxidized residue into a 2–10 nt flap which then gets cleaved by FEN1, and the resultant nick sealed by LIGI. Therefore, minimal reconstitution of LP-BER requires Pol β, APE1, FEN1, and LIGI.

**Figure 8.**
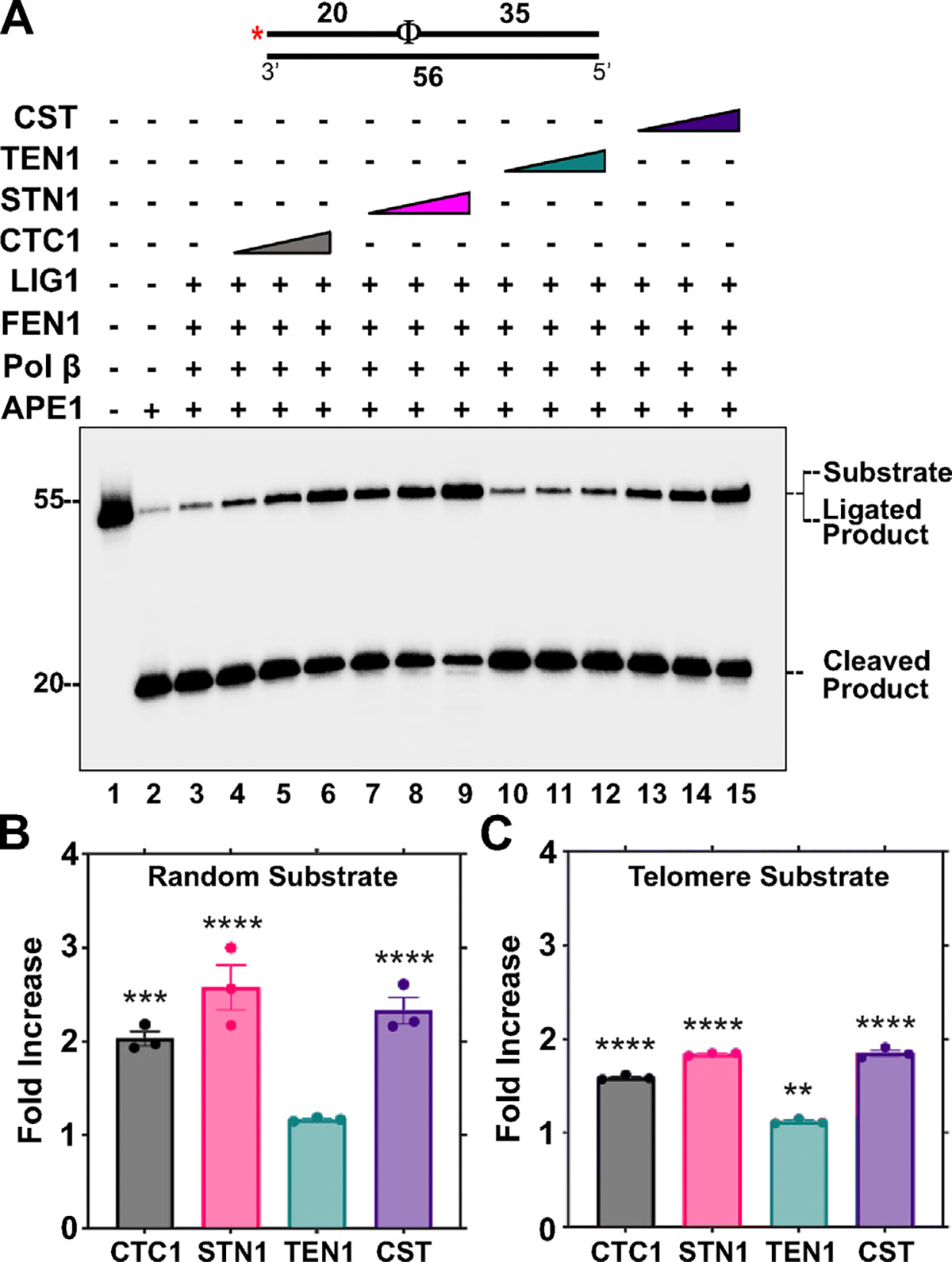
The CST complex, CTC1 and STN1 increase the efficiency of LP-BER. (A) The LP-BER pathway was reconstituted *in vitro* by combining APE1 (3 nM), Pol β (0.5 nM), FEN1 (0.01 nM), and LIGI (0.1 nM) on a random sequence abasic substrate containing a tetrahydrofuran (THF) residue (5 nM). An Φ represents a THF residue and a red asterisk represents the location of the ^32^P radiolabel on the substrate. The ligation efficiency was monitored following the addition of CTC1/STN1/TEN1 (100, 500, 1000 nM) or CST complex (14.75, 29.5, 59 nM) as denoted. Gel shown is representative of at least three replicate experiments. (B) Graph of the fold increase in ligation efficiency for the lanes containing the highest concentration of CST proteins utilized. (C) Shows the fold increase in ligation efficiency on a telomere substrate calculated as in (B). The error bars represent the standard error mean. The random substrate (U2:T1) and telomere substrate (U3:T2) was generated by annealing primers in a 1:2 ratio. (****p < 0.0001, **p < 0.01).

The BER components were co-incubated in the presence of increasing concentrations of CST or individual subunits on both non-telomeric and telomeric THF-containing substrates, resembling an abasic site. Our results demonstrate the ability of the full CST complex or individual subunits to stimulate the overall repair efficiency of LP-BER (Figure 8). On the random sequence substrate, there was a calculated fold increase in ligation efficiency of 2.2-fold for the entire complex, 2.0-fold for CTC1, and 2.5-fold for STN1 (Figure 8B). However, TEN1 alone did not simulate BER *in vitro*. Similar results were observed on the telomeric substrate although to a lesser extent (Figure 8C). Due to the presence of the radiolabel on the 5′end of the THF containing primer, we cannot eliminate the possibility of Pol β displacing the APE1 cleaved downstream primer and synthesizing till the end of the template. To confirm that the 55 nt product was from repair ligation and not due to full length synthesis by Pol β, we labeled the 3′end of the THF containing primer and assessed LP-BER efficiency using this substrate. Similar to the results in Figure 8, we observed both CTC1 and STN1 stimulated the ligation product, while TEN1 did not. The full CST complex was also able to stimulate repair thus confirming the role of CST in improving repair efficiency (Figure S5).

### Loss of CST increases cellular 8-oxoGs

**Figure 9.**
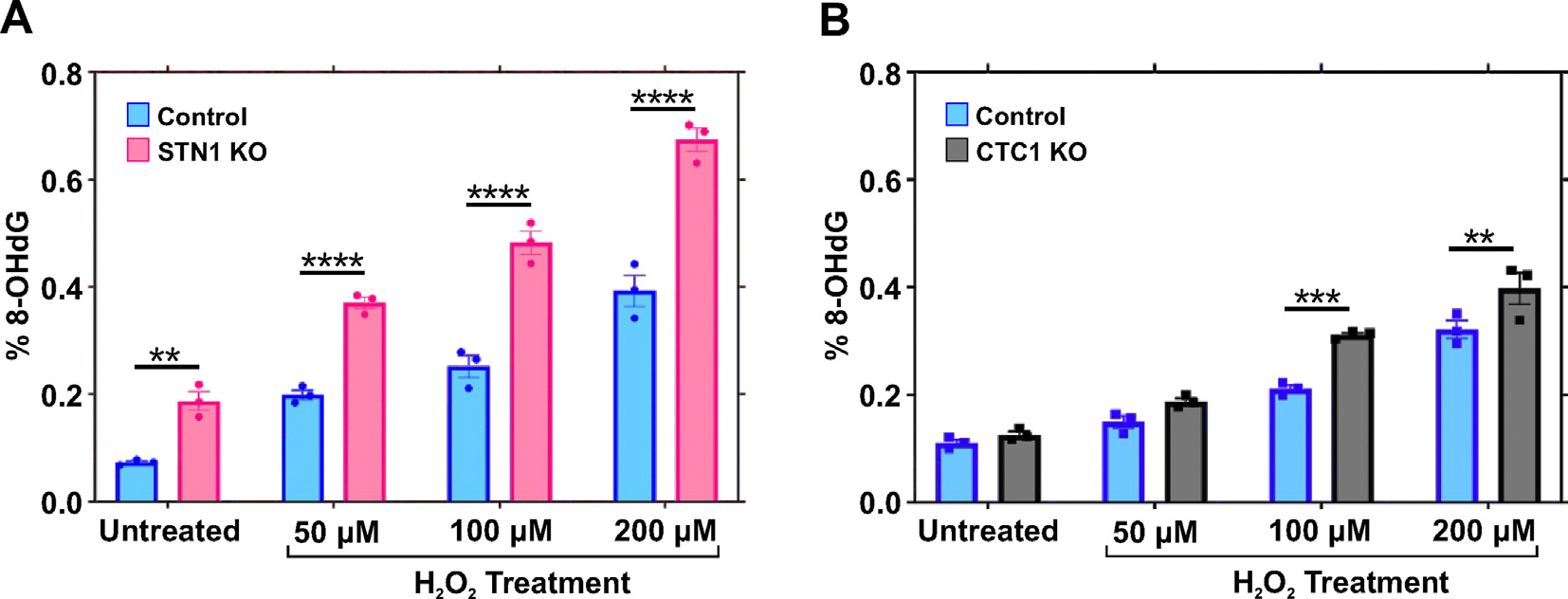
CST prevents the accumulation of genomic 8-oxo-Gs. Parental and STN1 KO (A) or CTC1 KO (B) cells were treated with 0, 50, 100 or 200 μM H_2_O_2_ for 1 h before release and recover for 4 h. Cells were collected, and genomic DNA extracted and 8-oxoG levels measured. A representative trial of at least two independent biological trials is shown. Circles represent three technical replicates. The error bars represent the standard error mean. (****p < 0.0001, ***p < 0.001, **p < 0.01).

Our *in vitro* biochemical reconstitution experiments suggest that CST plays an undiscovered role in the repair of oxidative DNA damage through BER. Therefore, we hypothesized that cells lacking CST would be compromised in their ability to repair oxidized bases. Based on our *in vitro* work showing that CST stimulates OGG1, Pol β, APE1, and LIG1, we predicted that there would be increased levels of oxidative DNA damage in CST deficient cells. As mentioned previously, 8-oxoG is the most common form of oxidative base damage and is highly mutagenic. Accordingly, we tested whether cells lacking STN1 (Figure S6) had increased levels of 8-oxoG. HeLa STN1 KO cells were treated with increasing concentrations of H_2_O_2_ (50, 100 or 200 μM) for 1 h. Cells were then washed and released into fresh media for 4 h prior to collection. Genomic DNA was then extracted and 8-oxoG content measured (Figure 9A). Intriguingly, even in the absence of exogenous oxidative stress, 8-oxoG levels in STN1 KO cells were approximately double that of the parental cells, indicating that CST aids in oxidative repair even in unstressed cells. Following H_2_O_2_ treatment, there was also a corresponding increase in the percentage of 8-oxoGs (Figure 9A).

To confirm our findings, we tested whether removal of CTC1 also increased the levels of 8-oxoG in a previously characterized HCT116 CTC1 KO cell line.^51,52^ While CTC1 loss significantly increased 8-oxoGs at higher concentrations of H_2_O_2_ (100 or 200 μM), its overall effect was much lower than STN1 loss. This may be due to differences in parental cell lines (i.e., STN1 KO – HeLa, CTC1 KO – HCT116). However, Western blot analysis of the cell lines revealed that, following STN1 KO, CTC1 and TEN1 levels are greatly diminished, while CTC1 KO did not affect the levels of STN1 or TEN1 (Figure S6). This suggest that STN1-TEN1 can positively affect 8-oxoG repair even in the absence of CTC1, whereas STN1 KO results in an overall loss of cellular CST, leading less repair. In both KO lines, Pol β levels are unaffected (Figure S6). These findings suggest that CST aids in the repair of oxidized bases and corroborate our *in vitro* studies that CST may promotes BER to minimize oxidative DNA damage.

## Discussion

Human CST is a ssDNA binding protein that is essential for telomere maintenance with additional roles in DNA replication and repair. Due to its role at telomeres,^20^ preference for G-rich DNA,^25^ ability to stimulate another polymerase (i.e., Pol a),^26,29,30^ and recent findings that CST deficiency increases oxidative DNA damage,^33,34^ we sought to characterize whether CST functions in BER by studying the biochemical interactions between CST and BER-associated proteins. Combined our results demonstrate that CST interacts with and stimulates the enzymatic activities of several BER-associated proteins, in particular the activities of Pol β. Furthermore, full reconstitution of LP-BER showed a direct stimulation by CST *in vitro*. Finally, an assessment of cellular 8-oxoG levels indicate that CST is needed to prevent the accumulation of basal endogenous and exogenous oxidative DNA damage. Combined, these studies provide a clear link between CST and oxidative DNA repair through the stimulation of BER.

The ability of CST and individual CST subunits to stimulate the different activities of Pol β, (i.e., DNA synthesis, strand-displacement, dRP lyase), portends a direct interaction that is independent of DNA. Consistent with this idea, we observe direct interaction with purified proteins (Figure S3) and co-immunoprecipitation in lysates treated with benzonase (Figure 1D). Considering that Pol β is the chief polymerase responsible for nucleotide synthesis during BER, our observed biochemical stimulation of Pol β by CST provides compelling evidence that CST stimulates Pol β to promote BER. This agrees with past work indicating that human CST acts as a stimulatory factor for Pol α.^26,28,29^ It is also interesting to note that individual CST subunits still stimulated Pol β (although to differing degrees), which suggests multiple contact points between CST and Pol β. This is in line with a recent structure of the CST-Pol α complex, which found interactions sites between all three subunits of CST and Pol α with the N-terminus of STN1 and TEN1 interacting with the primase domain and CTC1 and the C-terminus of STN1 interacting with the polymerase domain.^53^

**Figure 10.**
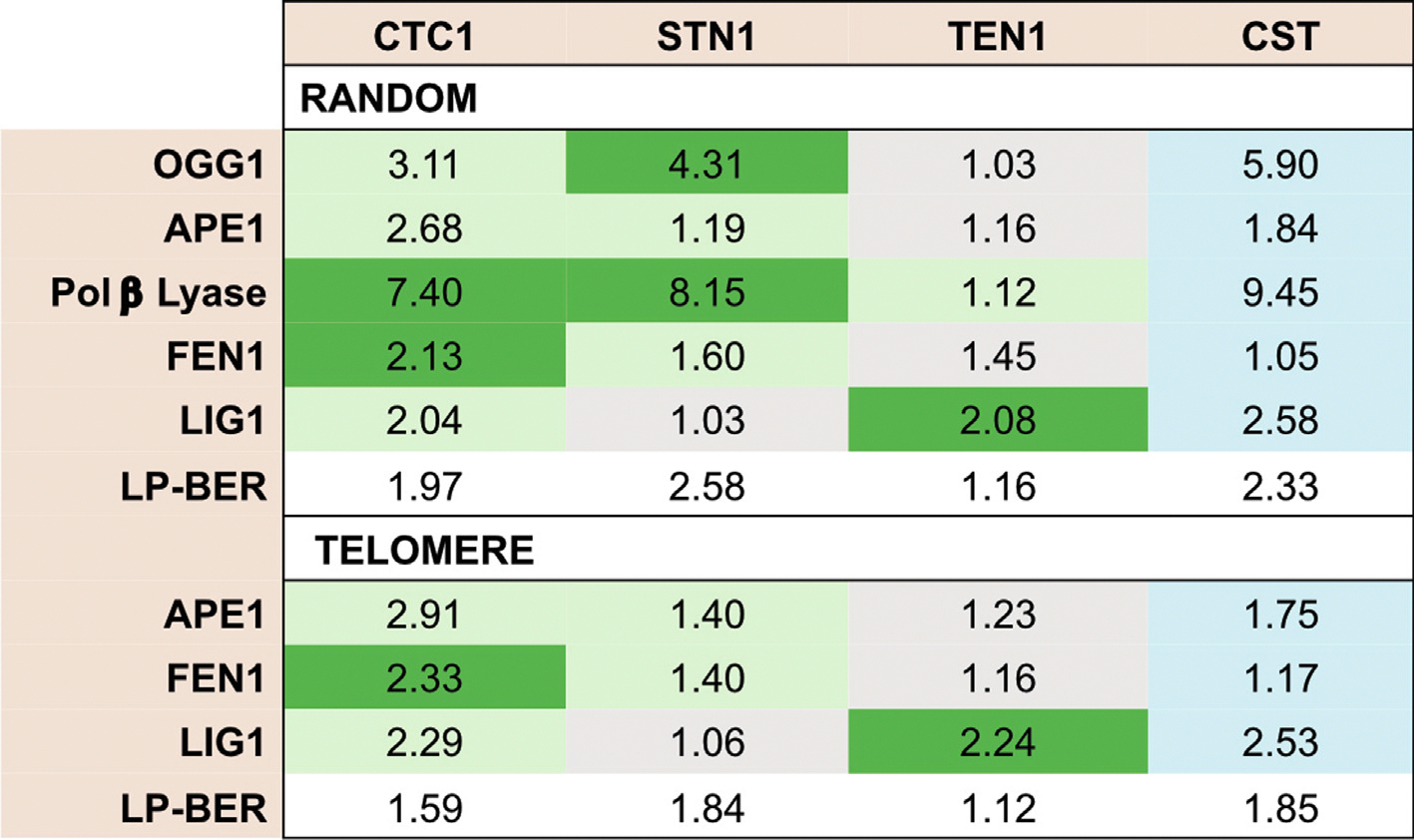
Summary of biochemical studies and interaction data. Calculated fold changes for each BER step tested on a random sequence or telomere sequences are shown. Interactions between individual subunits of the CST complex and the proteins in the BER pathway in the absence of DNA within cell lysates were tested using Co-IP from Figure 1D. Detection of a weak interaction (light green), strong interaction (dark green) or no interaction (grey) are denoted in each column. Interactions between recombinant proteins of the BER pathway (denoted in blue) and the CST complex were assessed using a binding assay in Figure S3.

Moreover, we note that CST interacted with and stimulated the enzymatic activities of other proteins in the pathway, including OGG1, APE1, FEN1, and LIG1. Therefore, CST may function more generally to stimulate the entire process. Initial characterization of mammalian CST focused on its ability to interact with and stimulate Pol α, a function conserved across eukaryotes.^54^ However, more recent work has shown that CST can also stimulate the activities of RAD51 and MRE11.^55,56^ These interactions are proposed to facilitate the rescue of stalled replication forks. Thus, CST likely acts at several points to facilitate or stimulate this process. In like manner, our results suggest that CST acts at multiple points to stimulate BER and prevent the accumulation of oxidative DNA damage. (A summary of fold-changes in activities of BER proteins in the presence of individual or the entire CST complex combined with Co-IP data are noted in Figure 10).

It is interesting to note that STN1 KO resulted in increased 8-oxoGs even in the absence of H_2_O_2_ treatment whereas CTC1 KO only increased at higher levels of H_2_O_2_ (Figure 9). This correlates with the diminished levels of CTC1 and TEN1 in the STN1 KO cells, which appears as an almost complete loss of CST from these cells (Figure S6). In contrast, CTC1 KO did not alter STN1 and TEN1 levels. This is not surprising given that STN1 bridges the interaction between CTC1 and TEN1 and expression of STN1 was previously reported to stabilize CTC1.^19,38,57^ However, this may provide some insight into how CST promotes BER. Our biochemical analysis revealed that the full CST complex can stimulate all steps of LP-BER *in vitro*, except for FEN1 activity, with the greatest effect on OGG1 and Pol β polymerase and lyase activities. Thus, in cases where CST is required for BER, its loss is predicted to have an immediate impact the recognition/removal of the oxidized base through the stimulation of OGG1. This is consistent with an overall loss of CST in the STN1 KO cells leading to increased 8-oxoGs even in the absence of exogenous oxidative damage. In contrast, removal of CTC1 causes only a minor increase in 8-oxoGs after exogenous oxidative DNA damage. Based on our *in vitro* data, STN1 strongly interacts with OGG1 and stimulates its activity as well as Pol β polymerase and lyase activities. Accordingly, BER may still proceed in the absence of CTC1 until 8-oxoGs reach levels that require the assistance of the full CST complex. Whether CST subcomplexes form in the cell is still currently unclear but it is likely that STN1-TEN1 can function in the absence of CTC1 similar to their yeast counterparts.^58,59^

While further work will be required to determine the timing and mechanism of CST localization to oxidative DNA damage, we speculate that CST acts to localize or recruit BER factors to G-rich regions of the genome, such as telomeres and CpG islands, where oxidative DNA damage is common and CST is known to localize and bind.^25,60^ At these sites, CST could help to resolve G4s, which may affect the efficiency of 8-oxoG repair in this structural motif.^61,62^ The presence of G4s may make it difficult for BER-associated proteins to optimally function, as has been observed for other proteins. For example, it has been shown that DNA Pol δ synthesis is severely impeded by the presence of G4s.^63^ Given that CST binds to G-rich telomeric sequences and can resolve G4 structures, CST is well-equipped to promote oxidative repair in G-rich DNA. It is important, however, to note that the stimulatory effects of CST on individual BER-associated proteins and full reconstitution of LP-BER was similar on both telomere and non-telomere substrates. Therefore, CST may function more generally in BER across the genome. A recent study by Hara et al. also demonstrated that STN1 depletion results defective S-phase progression following H_2_O_2_ and affected RAD51 loading.^33^ Thus, it is possible that the increase in 8-oxoGs observed relates to CST’s ability to load RAD51 at stalled replication forks. However, our biochemical work demonstrates a direct interaction and stimulation of BER and RAD51 does not appear to have a major role in BER, arguing against an indirect affect from replication fork stalling. Nevertheless, future work to tease out the diverse roles of CST in genome maintenance and sites of localization following different types of DNA damage will be important to fully understand CST’s role in BER as well as other repair pathways.

Overall, our biochemical results have identified previously unknown interactions between CST and BER-associated proteins and provide an important foundation for future studies seeking to understand how oxidative damaged DNA is repaired to maintain genome integrity and prevent disease.

## Materials and Methods

### Recombinant proteins

Recombinant human Pol β,^64^ FEN1,^65^ APE1,^66^ and LIG1,^67^ were purified as described previously. Recombinant human OGG1 was commercially purchased from Novus Biologicals (NBP1-45318). All recombinant BER proteins contain a 6X-His tag. Constructs for purification of CST as well as the individual CTC1, STN1 or TEN1 subunits were kindly provided by Dr. Carolyn Price from the University of Cincinnati.

#### Human FLAG-CTC1.

The construct for full-length FLAG-tagged human CTC1 was previously generated by cloning the DNA sequence for the CTC1 gene into the pcDNA3.1 vector. Transfection of pcDNA-FLAG-CTC1 plasmid DNA was performed in Expi293 cells using the ExpiFectamine kit according to manufacturer protocol (Thermo Fisher Scientific). Cells were harvested 72 h after transfection.

Cell lysates were prepared by sonicating the thawed cell pellet with 5 s on/off pulses for 10 min at 35% amplitude in lysis buffer containing 25 mM Na-HEPES, pH 7.5, 75 mM NaCl, 0.5 mM EDTA, 0.5% Triton X-100, 10% glycerol, 0.5 mM DTT, 1 mM PMSF, and protease inhibitor cocktail tablet (Roche Applied Science). The clarified cell lysate was collected by centrifugation at 3000*g* for 20 min and incubated with 100 μL FLAG M2 agarose beads (Sigma-Aldrich) for 2 h on a LabQuake rotator. Anti-FLAG M2 agarose beads were prepared for use according to the protocol published by Gerace and Moazed.^68^

After binding, the beads were washed thrice in buffer containing 25 mM Na-HEPES, pH 7.5, 75 mM NaCl, and 0.5 mM EDTA, 5% glycerol, and 1 mM PMSF. The elution step was performed by addition of elution buffer containing 200 μg 3X FLAG-peptide (MedChemExpress), 25 mM Na-HEPES, pH 7.5, 50 mM NaCl, and 1 mM PMSF. Peak fractions containing purified CTC1 protein were confirmed using SDS-PAGE and Western Blotting with α-FLAG (Sigma-Aldrich) and α-CTC1^52^ antibodies. CTC1 protein concentration was assessed using QuBit protein assay on a QuBit Fluorometer (Life Technologies). The purified CTC1 fractions were supplemented with 25% glycerol and 0.5 mg/mL BSA, aliquoted, and kept frozen at −80 °C. Due to stability concerns, aliquots of CTC1 were only thawed twice for use in functional assays. As shown in Figure S2A, right, repeated freeze–thaw cycles resulted in the degradation of the CTC1 protein.

#### Human GST-STN1.

Full-length GST-tagged human STN1 was cloned into the pGEX-6p-3 plasmid and purified as an N-terminal GST fusion via batch purification. The expression plasmid was introduced into *E. coli* strain BL21 (DE3) and allowed to grow at 25 °C shaking for 220 RPM overnight following induction with 0.1 mM IPTG. Bacterial pellets were collected by centrifuging at 12,000*g* for 30 min. Pellets were then kept frozen at −80 °C. Pellets from 1 L of culture were resuspended in 25 mL ice cold lysis buffer containing 10% glycerol, 1 mM PMSF, 1 mM 2-mercaptoethanol, 0.5 mg/mL lysozyme, and protease inhibitor cocktail tablet, and kept on ice for 20 min before sonicating using 30 s on/off pulses at 35% amplitude for 10 min. Lysates were diluted in 1X PBS buffer to 40 mL and clarified by centrifuging at 10,000*g* for 20 min. Glutathione sepharose resin (GE Healthcare) was equilibrated in 1X PBS buffer and allowed to incubate with the cleared lysate for 2 h at 4 °C. Beads were washed 3X with 20 mL 1X PBS buffer, and 1X with 10 mL wash buffer containing 50 mM Tris–HCl, pH 8.0, and 150 mM. Washed beads were resuspended in 2 mL of elution buffer containing 50 mM Tris–HCl, pH 8.0, 150 mM NaCl, and 20 mM L-glutathione reduced, pH 8.0, and allowed to rotate for 20 min at 4 °C before fraction collection. The presence of purified GST-STN1 was confirmed by SDS-PAGE and the protein concentration assessed by the QuBit protein assay. Aliquoted fractions were frozen and stored at −80 °C.

#### Human His-TEN1.

Full-length His-tagged human TEN1 was cloned into pET28a, cultured in BL21 (DE3) competent *E. coli* cells, and expressed at 37 °C shaking overnight following induction with 0.1 mM IPTG. Cell pellets were collected via centrifugation at 12,000*g* and then kept frozen at −80 °C. Pellets were resuspended in ice cold lysis buffer at the ratio of 1 mL buffer per 1 g of pellet. The lysis buffer consisted of 20 mM Tris–HCl, pH 7.5, 0.5 M NaCl, 30 mM imidazole, pH 7.4, 1 mM DTT, 0.01% Igepal CA-630, 6 M guanidine-HCl, and 1 protease inhibitor cocktail tablet. Lysates were sonicated using 15 s on/off pulse cycles for 8 min at 40% amplitude and loaded onto a pre-equilibrated Ni-NTA agarose resin (Qiagen). Using an NGC chromatography system (Bio-Rad), the resin was washed using 10X column volumes of binding buffer containing 20 mM Tris–HCl, pH 7.5, 0.5 M NaCl, 30 mM imidazole, pH 7.4, 1 mM DTT, 0.01% Igepal CA-630, and a protease inhibitor cocktail tablet (Roche Applied Science). Purified TEN1 was collected after gradient elution in buffer containing 20 mM Tris–HCl, pH 7.5, 0.5 M NaCl, and 500 mM imidazole, pH 7.4. Peak fractions containing TEN1 were pooled and concentrated using Amicon Ultra centrifugal filters, assessed by SDS-PAGE and QuBit Fluorometer. Aliquoted fractions were frozen and stored at −80 °C.

#### Human CST complex.

HEK293T cells were co-transfected with pcDNA3.1-FLAG-CTC1, pcDNA3.1-myc-STN1, and pcMV-Sport6-TEN1 in a ratio of 2:1:1, using polyethylenimine (PEI) (Polysciences). After 72 h, cells were harvested, suspended in 1X PBS, pelleted via centrifugation at 2000*g* for 20 min, and then stored at −80 °C. Cell pellets were lysed in NETN buffer (40 mM Tris–HCl, pH 8.0, 100 mM NaCl, 1 mM EDTA, 0.5% NP40, 1X protease inhibitors [1 μg/ml pepstatin A, 5 μg/ml leupeptin, 1 μg/ml E64, 2 μg/ml aprotinin, and 5 μg/ml antipain], and 1X phosphatase inhibitors [4 mM β-glycerophosphate, 4 mM sodium vanadate, and 20 mM sodium fluoride]). Cell lysates were centrifuged at 15,000*g* for 20 min at 4 °C. The supernatant was run on a column of single-stranded DNA beads (Bethesda Research Laboratories, Inc.) followed by 5 column volume (CV) washes of NETN, 5 CV BC75 (20 mM Tris–HCl, pH 8.0, 75 mM NaCl, 0.2 mM EDTA, 10% glycerol) and BC200 (20 mM Tris–HCl, pH 8.0, 200 mM NaCl, 0.2 mM EDTA, 10% glycerol). CST was eluted with 3 CV BC500 (20 mM Tris–HCl, pH 8.0, 500 mM NaCl, 0.2 mM EDTA, 10% glycerol, 100 μg/mL BSA) and 3 CV BC1000 (20 mM Tris–HCl, pH 8.0, 1000 mM NaCl, 0.2 mM EDTA, 10% glycerol, 100 μg/mL BSA). DNA binding activity was checked by EMSA on a 48 nt substrate and elutions with active ssDNA binding activity were combined and incubated overnight with 1 ml of 50% M2 α-Flag (Sigma-Aldrich) agarose bead slurry. Beads were washed with at least 3 CV of NETN before eluting CST using storage buffer (20 mM Tris–HCl, pH 8.0, 50 mM NaCl, 0.5 mM EDTA, 0.25% NP40, 10% glycerol, 100 μg/mL BSA and 100 μg/mL 3x FLAG peptide [Sigma-Aldrich]).

### Synthetic oligonucleotides

All custom synthetic oligonucleotides used for *in vitro* experiments were designed and ordered from IDT (Integrated DNA Technologies, Coralville, IA), except the T1 oligonucleotide which was purchased and prepared by Midland Certified Reagent Company. The sequences are listed in Table S1. “G*” denotes an 8-oxoG, “Φ” denotes a tetrahydrofuran (THF) residue, and “p” represents a 5′ phosphorylation modification. Upstream and downstream primer sequences are listed in the 5′–3′ direction, while the template sequences are shown in the 3′–5′ direction to help with orientation. Primers were labeled as denoted in the figures either at the 5′ end using [γ-^32^P]ATP and T4 polynucleotide kinase (Roche Applied Science), or the 3′ end using [α-^32^P]dCTP and the Klenow fragment of *E. coli* DNA polymerase I (Roche Applied Science). All radionucleotides were purchased from PerkinElmer Life Sciences. For 3′-end labeling, the primer was annealed to the template containing a G-overhang on the 5′ end to facilitate the incorporation of [α-^32^P]dC TP by Klenow. The labeled primers were isolated and purified on a 15% polyacrylamide, 7 M urea denaturing gel. The DNA was then ethanol precipitated and quantified using a Beckman Coulter Liquid Scintillation Counter. Primers were annealed in a 1:2:4 ratio of upstream primer to template to downstream primer in nuclease-free duplex buffer (Integrated DNA Technologies) by heating at 95 °C for 5 min, then cooling slowing to room temperature overnight. Substrates without a downstream sequence were annealed in a 1:2 ratio of upstream primer to template.

### Enzyme assays

Enzymatic assays were performed by generating 20 μL reaction mixtures containing 5 nM ^32^P-radiolabeled DNA substrate and the enzyme of interest (OGG1, Pol β, APE1, LIG1, or FEN1) along with the addition of the full CST complex and/or individual subunits (CTC1, STN1, TEN1) at the concentrations listed in the figure legends. Reactions were incubated at 37 °C for 10 min, unless otherwise noted. The Pol β reaction buffer consisted of 50 mM Tris–HCl, 2 mM DTT, 2 μg/mL BSA, 2 mM ATP 8 mM MgCl_2_, 25 nM NaCl, and 0.1 mM dNTP mixture (Roche Applied Science). The OGG1/APE1/FEN1 reaction buffer consisted of 50 mM Tris–HCl, pH 8.0, 2 mM DTT, 0.25 mg/mL BSA, 30 mM NaCl, 5% glycerol, 8 mM MgCl_2_, and 2 mM ATP. The LIG1 reaction buffer consisted of the Pol β reaction buffer without dNTPs. Reactions were terminated using 2X termination loading dye consisting of 90% formamide (v/v), 10 mM EDTA, 0.1% bromophenol blue, and 0.1% xylene cyanole. After termination, reaction mixtures were boiled for 5 min at 95 °C, loaded onto a pre-heated 12% polyacrylamide urea denaturing gel, and separated by electrophoresis at 80 Watts.

### Lyase activity assay

The dRP lyase activity of Pol β was assessed by measuring the excision of the 5′-dRP residue on a duplex DNA substrate containing an uracil residue at position 20 (U5:T3). The 5′-dRP residue was enzymatically generated by treating 5 nM of substrate with 1 U of Uracil DNA Glycosylase enzyme (New England Biolabs, M0280S) in 1X UDG Reaction Buffer (New England Biolabs, M0280) for 30 min at 37 °C. Next, the UDG cleaved substrate was incubated for 10 min at 37 °C with APE1 (100 nM) in reaction buffer containing 50 mM Tris–HCl, 2 mM DTT, 2 μg/mL BSA, 2 mM ATP 8 mM MgCl_2_, and 25 nM NaCl. Pol β (0.25 nM) along with the CST complex (5, 15, 45 nM) or subunits CTC1/STN1/TEN1 (250, 750, 1500 nM) were then added to the APE1 cleaved DNA and allowed to incubate for 10 min at 37 °C. Each reaction was 20 μL total. Following incubation, 3.4 μL of 2 M NaBH_4_ was added to each reaction to stabilize the dRP product, and the reactions were kept on ice for 30 min. Finally, each of the reaction samples were ethanol precipitated (this is essential to prevent smearing on the gel due to the NaBH_4_), separated by electrophoresis for 2.5 h on a 20% polyacrylamide gel at 80 W, dried and imaged via PhosphoImaging.

### LP-BER assay

The LP-BER pathway was reconstituted *in vitro* by incubating APE1, Pol β, FEN1, and LIGI with 5 nM of an abasic substrate containing a THF residue in buffer containing 50 mM Tris–HCl, 2 mM DTT, 2 μg/mL BSA, 2 mM ATP 8 mM MgCl_2_, 25 nM NaCl, and 0.1 mM dNTP mixture (Roche Applied Science). Reactions were incubated at 37 °C for 10 min and subsequently terminated samples were loaded onto a pre-heated 12% polyacrylamide urea denaturing gel and separated by electrophoresis at 80 Watts and ligation efficiency was monitored.

### Gel analysis

Following electrophoresis, gels were transferred to Whatman filter paper, wrapped in plastic wrap, and dried for 1 h on Bio-Rad vacuum gel drier. Reaction products were visualized by exposing the dried gels to phosphor screens overnight and scanning on a Typhoon 9500 phosphoimager (GE Healthcare). Gels displayed in figures are representative of at least three independent experiments and were analyzed using ImageQuant TL version 8.1. Quantitation of gels was performed using Image Studio version 5.2, and intensity traces were generated using ImageJ version 1.53, graphs were plotted using GraphPad Prism software. APE1 and FEN1 cleavage products were calculated using the following calculations: % Cleaved product: {(b)/(b + a)} × 100 (where, “b” is the cleaved product, and “a” is the remaining uncleaved substrate). Ligation products were calculated using the following calculations: {(b)/(b + a)} × 100 (where, “b” is the ligated product, and “a” is the remaining unligated substrate). Fold changes were calculated using the following calculation {(b)/(a)}, where “b” is the percentage activity of a BER enzyme in the presence of either individual subunits of the CST complex or the entire CST complex and “a” is the percentage activity of the BER enzyme by itself.

### Cell culture

HeLa 1.2.11 cells were maintained in RPMI 1640 media, HeLa iCas9 and HEK 293 T in DMEM, and HCT116 cells in McCoy’s media. All cells were grown at 37 °C with 5% CO_2_ and were supplemented with 10% fetal bovine serum and 1% penicillin/streptomycin. Cell lines were regularly checked for mycoplasma contamination. HeLa 1.2.11 cells were used for PLA analysis, HEK 293 T cells for the purification of the full CST complex and HeLa iCas9 and HCT116 cells for 8-oxoG analysis. HeLa iCas9 (inducible Cas9) cells were generously provided by Dr. Iain Cheeseman from Massachusetts Institute of Technology. STN1 inducible knockout (KO) cell lines were constructed following the protocol outlined by McKinley,^69^ using the sgRNA (5′ GGG GAC ACG ATC CGA GTC AGA 3′) to STN1. The sgRNA was cloned into pLenti-sgRNA using the BsmB1 restriction site, lentivirus prepared, and HeLa iCas9 cells transduced with the resultant virus. Clones were selected with puromycin and immunoblotting conducted to ensure successful KO. To induce the conditional gene KO of STN1, cells were incubated with 1 μg/ml Doxycycline (Dox) for 3–4 days. HCT116 CTC1^F/F^ cells have been previously described.^51^ Conditional CTC1 KO was induced by the addition of 10 nM Tamoxifen, as previously described. 8-oxoG detection was performed on day 15 after conditional deletion of either STN1 or CTC1.

### Co-immunoprecipitation

HEK293T cells were transfected with either pcDNA3.1-FLAG-CTC1, pcDNA3.1-myc-STN1, or pcDNA3.1-FLAG-TEN1 (Genscript, OHu29186), using polyethylenimine (PEI) (Polysciences). After 24 h, transfected cells were left untreated or treated with 200 μM of H_2_O_2_ for a period of two hours to induce oxidative damage. Following two hours of treatment, the cells were washed with 1X PBS, harvested and lysed in RIPA buffer. Lysates were treated with 20U of benzonase (Millipore, ≥250 units/μL) for two hours. Immunoprecipitation was performed according to the Dynabeads protein G manual (Thermo Fisher Scientific) manufacturers protocol with minor modifications. Briefly, 1.0 μg of antibodies to OGG1 (Invitrogen, PA1-31402), APE1 (Cell Signaling, 4128), Pol β (Abcam, ab175197), FEN1 (Abcam, ab17994), LigI (Thermo Fisher 18051-1-AP), and control IgG (SantaCruz Biotech, sc-2027) were prebound to 1 mg of HEK293T cells lysates (untreated or treated) with 200 μl of 1X PBST for 1 h at room temperature with end-over mixing. 50 μL of Dynabeads were prepared and antibody bound cell lysate were incubated with end-over mixing overnight. The Dynabeads-antibody-antigen complex was washed with 200 μL of wash buffer and separated on the magnet between the washes. Bound proteins were eluted using 20 μL of elution buffer and 20 μL of 2X NuPAGE LDS sample buffer with NuPAGE sample reducing agent (Invitrogen) followed by heating the samples at 70 °C for 10 mins. The immunoprecipitates (20 μL for CTC1 and STN1 and 40 μL for TEN1) were loaded on a gradient 4–15% precast TGX gels (Bio-rad). Immunoblotting was performed using a 1:5000 dilution of anti-FLAG (Sigma, A8592) or anti-Myc (Abcam, ab223895) antibodies. Blots were visualized using the Typhoon 5 Imager and quantified by densitometry using LI-COR Image Studio Lite Ver 5.2.

### Whole cell protein extraction

Cell pellets were lysed, sonicated and nuclease-treated, as previously described.^52^ The supernatant was collected, and protein concentration measured by BCA assay (Thermo Fisher Scientific). The samples were then mixed with SDS-PAGE loading buffer and analyzed by Western blot, as previously described.^52^ Primary: OBFC1 (STN1) (Novus, NBP2-01006), CTC1,^51^ TEN1,^70^ Pol β, (Abbexa, abx304879), α-Actinin (Santa Cruz, SC17829). Secondary: goat-anti-mouse-HRP (Thermo Scientific, 32430), goat-anti-rabbit-HRP (Thermo Scientific, 32460).

### Proximity Ligation Assay (PLA)

HeLa 1.2.11 cells were fixed for 20 min at room temperature with 4% formaldehyde in 1X PBS followed by permeabilization with 100% methanol for 20 min at −20 °C·H_2_O_2_ treated cells were incubated with 200 μM H_2_O_2_ for 2 h prior to fixation. Subsequent steps were performed with the Duolink PLA kit (Millipore-Sigma) as previously described,^35^ except the first wash after primary incubation was performed using wash buffer A. Primary antibodies: 1:100 mouse α-STN1 (Novus, NBP2-01006), 1:100 rabbit α-POLB (Abbexa, abx304879), 1:100 rabbit α-PolA1 (Bethyl Laboratories, A302-850A), 1:100 rabbit α-XRCC1 (Genetex, GTX111712), 1:100 mouse α-XRCC1 (Invitrogen, MA1-12640), 1:100 rabbit α-PARP1 (Proteintech, 6520-1-Ig), 1:100 rabbit α-OGG1 (Proteintech, 15125-1-AP). Images were taken on an EVOS FL microscope, using a 40X objective (Thermo Fisher Scientific). Ten images were scored per independent, biological experiment for each condition. Image analysis was performed with CellProfiler. Violin plots and p-values were generated with GraphPad Prism.

### Detection of 8-oxo-Gs

Genomic DNA from HeLa iCas9 sgSTN1 cells (CTRL – no Doxycycline, KO – Doxycycline reated) or HCT116 CTC1^F/F^ cells (CTRL – no Tamoxifen, KO – Tamoxifen treated) with or without H_2_O_2_ treatment was collected and analyzed for the level of 8-oxo-Gs. Cells were treated for 1 h with the indicated amount of H_2_O_2_, washed three times with 1X PBS, and released into fresh media for 4 h. Cells were then collected and genomic DNA isolated (Thermo Scientific, K0722). 8-oxoGs were then measured using an ELISA-based EpiQuik 8-OHdG kit (Epigentek, P-6004-96), per manufacturer’s instructions.

### Statistics

Error bars indicate the ±standard error mean (SEM) of at least three independent biological experiments. Dots in bar graphs represent independent biological replicates, except for Figure 9 which represent technical replicates and are representative of at least two independent biological replicates. *P*-values were calculated in GraphPad Prism using an unpaired, two-tailed Mann-Whitney test for the PLA data in Figures 1B, C and S1 and, for graphs in Figures 2–3 and 5–9, a two-way ANOVA test using multiple comparisons to compare the column means to the control lacking CST or individual CST subunits.

## Supplementary Material

Supplemental Materials

## Data Availability

Data will be made available on request.
