# Supplementary material for: Human CST Stimulates Base Excision Repair to Prevent the Accumulation of Oxidative DNA Damage
Source: J Mol Biol. Author manuscript; Available in PMC 2025 Feb 26. (PMC11864811; doi:10.1016/j.jmb.2024.168672)
Supplement: Supplemental Materials [file NIHMS2054208-supplement-Supplemental_Materials.docx]

**SUPPLEMENTAL DATA**

**HUMAN CST STIMULATES BASE EXCISION REPAIR TO PREVENT THE ACCUMULATION OF OXIDATIVE DNA DAMAGE**

^1^Brandon C. Wysong, ^2^P. Logan Schuck, ^1^Madhumita Sridharan, ^1^Sophie Carrison, ^1^Yuichihiro Murakami, ^1,^ **^§^**Lata Balakrishnan, ^2,3^ **^§^**Jason A. Stewart

Affiliation:

^1^Department of Biology, School of Science, Indiana University, Indianapolis, Indiana, USA; ^2^Department of Biological Sciences, University of South Carolina, Columbia, USA; ^3^Department of Biology, Western Kentucky University, Bowling Green, KY, USA

Corresponding Authors:

**^§^**To whom correspondence should be addressed: Lata Balakrishnan, Department of Biology, Indiana University, Indianapolis, IN 46202, Phone: (317) 274 1290; Email: [latabala@iu.edu](mailto:latabala@iu.edu); Jason A. Stewart, Department of Biology, Western Kentucky University, Bowling Green, KY, Email: [jason.stewart@wku.edu](mailto:jason.stewart@wku.edu)

**List of Data:**

**Figure S1. STN1 associates with PARP1 and XRCC1.**

**Figure S2. CST complex and subunit purification and DNA binding activity.**

**Figure S3. CST interacts with BER components *in vitro*.**

**Figure S4. CST complex and individual subunits stimulate Pol β synthesis.**

**Figure S5. The CST complex, CTC1 and STN1 increase the efficiency of LP-BER.**

**Figure S6. CST protein levels in STN1 KO and CTC1 KO cells.**

**Table S1. List of oligonucleotide substrates**

**Figure S1. *STN1 associates with PARP1 and XRCC1.*** (A) Proximity ligation assay (PLA) performed in HeLa cells with antibodies to STN1, PARP1 or in combination (STN1+ PARP1). (B) PLA was performed in HeLa cells as in (A) with antibodies to STN1 and XRCC1. (C) PLA was performed as in (A) with antibodies to Pol β and XRCC1. Violin plots of PLA foci per nucleus. n = 4 independent, biological experiments for A-C. Bold dashed line: median, dashed lines: first and third quartiles. (****P < 0.0001).

**Figure S2. *CST complex and subunit purification and DNA binding activity.*** Recombinant proteins were purified as described in Materials and Methods (A) Left, Coomassie blue stained SDS-PAGE gel (4-15% gradient gel) of purified recombinant proteins. Right, Coomassie blue stained SDS-PAGE gel (4-20% gradient gel) of purified CTC1 protein after repeated freeze-thaw cycles. * indicates CTC1 degradation products, which were confirmed using an anti-FLAG antibody. (B) 5 nM of a 20 nt ssDNA substrate (5' IR-label) was bound with increasing concentrations (500 and 1000 nM) of CTC1, STN1 and TEN1 and (15 and 30 nM of CST complex). Substrate bound products were separated by 6% native PAGE gel and analyzed. Substrate alone and protein-bound substrate are indicated on the gel.

***
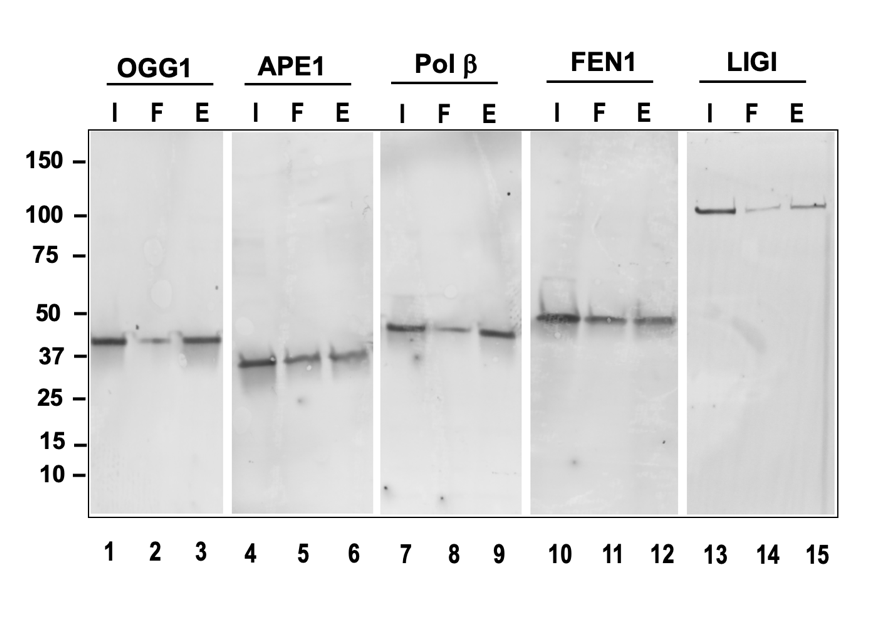
***

**Figure S3. *CST interacts with BER components in vitro.***

Recombinant individual BER proteins (OGG1, APE1, Pol β, FEN1 and LIGI) were incubated with the CST complex in a 1:1 ratio for 2 hours on ice in a coupling buffer (25 mM HEPES (pH 7.5), 100 mM NaCl, 1 mM EDTA, 1 mM dithiothreitol, and 10% glycerol). Following this initial incubation, the coupling reactions were transferred to 50 µL of M2 FLAG agarose beads and further incubated for 4 hours in cold with end over rotation on a LabQuake rotator. After binding, the beads were washed thrice in buffer containing 25 mM Na-HEPES, pH 7.5, 75 mM NaCl, and 0.5 mM EDTA, 5% glycerol, and 1 mM PMSF. The elution step was performed by addition of elution buffer containing 200 µg 3X FLAG-peptide (MedChemExpress), (25 mM Na-HEPES, pH 7.5, 50 mM NaCl, and 1 mM PMSF). 50% of recombinant protein input (I), flowthrough from washes (F) and eluate (E) were separated on a 4-15% gradient SDS-PAGE gel and immunoblotted using an anti-6X-His (iFlour 647) (Genscript, A01802).

**Figure S4. *The CST complex, CTC1 and STN1 increase the efficiency of LP-BER.*** (A) Synthesis by DNA Pol β (10 nM) on a 6 nt gap random sequence substrate (5 nM) in the presence of increasing amounts of CTC1/STN1/TEN1 (100, 500, 1000 nM) or CST (5, 15, 30 nM) as denoted in the figure. The red asterisk shown on the substrate in the figure represents the location of the ^32^P radiolabel. Gel shown is representative of at least three independent experiments. (B) The trace at the right of the gel depicts the signal intensity of the synthesis products in the lanes containing the highest concentration of CTC1(red trace)/STN1 (purple trace)/TEN1 (orange trace)/CST (green trace) [lanes 6, 10, 14, and 18] as compared to Pol β synthesis alone (black trace) [lane 2]. (C) Traces from a gel with a 6 nt gap telomere sequence substrate calculated as in (B). (D) Synthesis by DNA Pol β (10 nM) on a synthesis substrate (5 nM) in the presence of increasing amounts of CTC1/STN1/TEN1 (100, 500, 1000 nM) or CST (5, 15, 30 nM) as denoted in the figure. The red asterisk shown on the substrate in the figure represents the location of the ^32^P radiolabel. Gel shown is representative of at least three independent experiments. (E) The trace at the right of the gel depicts the signal intensity of the synthesis products in the lanes containing the highest concentration of CTC1(red trace)/STN1 (purple trace)/TEN1 (orange trace)/CST (green trace) [lanes 6, 10, 14, and 18] as compared to Pol β synthesis alone (black trace) [lane 2]. (F) Traces from a gel with a telomere sequence substrate calculated as in (E). The random substrate (U5:T3:D2) and telomere substrate (U6:T4:D3) was generated by annealing primers in a 1:2:4 ratio for A-C and random substrate (U5:T3) and telomere substrate (U6:T4) was generated by annealing primers in a 1:2 ratio for D-F.

**Figure S5. *The CST complex, CTC1 and STN1 increase the efficiency of LP-BER.*** The LP-BER pathway was reconstituted *in vitro* by combining APE1 (3 nM), Pol β (0.5 nM), FEN1 (0.01 nM), and LIGI (0.1 nM) on a random sequence abasic substrate containing a tetrahydrofuran (THF) residue (5 nM), as in Figure 9 except with the radiolabel on the 3ʹ-end. An Φ represents a THF residue and a red asterisk represents the location of the ^32^P radiolabel on the substrate. The substrate (U2:T1) was generated by annealing primers in a 1:2:4 ratio The ligation efficiency was monitored following the addition of CTC1/STN1/TEN1 (100, 500, 1000 nM) or CST complex (14.75, 29.5, 59 nM) as denoted.

**Figure S6. *CST protein levels in STN1 KO and CTC1 KO cells.*** Western blot analysis of the indicated proteins on day 15 after conditional deletion of STN1 (A) or CTC1 (B). Control indicates the parental cell lines without the induction of gene disruption (see Materials and Methods for additional details). *denotes a non-specific band; KO: knockout

| Primer | Length (in nt) | Sequence |
| --- | --- | --- |
| Upstream |  |  |
| U1 | 55 | 5’CGACCGTGCCAGCCTAAAAG*ACTTGCCCGTGCCACCATCCCGACGCCACCTCCTG-3’ |
| U2 | 55 | 5’-CGACCGTGCCAGCCTAAAAΦACTTGCCCGTGCCACCATCCCGACGCCACCTCCTG-3’ |
| U3 | 55 | 5’-CGACCGTGCCTTAGGGTTAGGGTTAGGGTTAΦGGTAAGGGTTAGGGCCACCTCCTG-3’ |
| U4 | 19 | 5’-CGACCGTGCCAGCCTAAAAC-3’ |
| U5 | 44 | 5’-GTCCACCCGACGCCACCTCCTGCCTTCAATGTGCTGGGATCCTA-3’ |
| U6 | 20 | 5’-GTCCGATCATCTATATCGAG-3’ |
| U7 | 55 | 5’-CGACCGTGCCAGCCTAAAAUACTTGCCCGTGCCACCATCCCGACGCCACCTCCTG-3’ |
| U8 | 26 | 5’-CGCCAGGGTTTTCCCAGTCACGACCA-3’ |
| U9 | 41 | 5’CTAGAACTGAGTGCCAGATAGCATTAGGGTTAGGGTTAGGG3’ |
| U10 | 20 | 5’ CGA CCG TGC CAG CCT AAA AC 3’ |
| U11 | 22 | 5’ CGA CCG TGC CTT AGG GTT AGG G 3’ |
| Downstream |  |  |
| D1 | 36 | 5’ΦACTTGCCCGTGCCACCATCCCGACGCCACCTCCTG-3’ |
| D2 | 60 | 5’-AGACGAATTCCGGATACGACGGCCAGTGCCGACCGTGCCAGCCTAAATTTCAATCC  ACCC-3’ |
| D3 | 20 | 5’-GTGACTGGAGTAGAGATGAC-3’ |
| D4 | 49 | 5’-GCCCAGTCACGTCGTTGTAAAACGGGTCGTGACTGGGAAAACCCTGGCG-3’ |
| D5 | 57 | 5’GGGTTAGGGAGGGTTAGGGTTAGGGTTAGGGTCCGATCGCCAGTCGCGTGCCTAGCG-3’ |
| D6 | 35 | 5’-pACTTGCCCGTGCCACCATCCCGACGCCACCTCCTG-3’ |
| D7 | 34 | 5’-pTTAGGGTTAGGGTTAGGGTTAGGGCCACCTCCTG-3’ |
| Template |  |  |
| T1 | 56 | 3’GCTGGCACGGTCGGATTTTGTGAACGGGCACGGTGGTAGGGCTGCGGTGGAGGACG-5’ |
| T2 | 57 | 3’-GCTGGCACGCAATCCCAATCCCAATCCCAATCCCAATCCCAATCCCGGTGGAGGAC  G-5’ |
| T3 | 110 | 3’-CAGGTGGGCTGCGGTGGAGGACGGAAGTTACACGACCCTAGGATGTTGGTTCTGC  TTAAGGCCTATGCTGCCGGTCACGGCTGGCACGGTCGGATTTAAAGTTAGGTGGG-5’ |
| T4 | 76 | 3’CAGGCTAGTAGATATAGCTCGGGATTGGGATTGGGATTGGGATTGGGATTGGGATTCA CTGACCTCATCTCTACTG-5’ |
| T5 | 58 | 3’GGGTCAGTGCAGCAACATTTTGCTGCCGGTCACTTAATTGATTGTCCGCACTTTGCCC-5’ |
| T6 | 86 | 3’GATCTTGACTCACGGTCTATCGTAATCCCAATCCCAATCCCAATCCCAATCCCAATCCCAGGCTAGCGGTCAGCGCACGGATCGCG-5’ |
| T7 | 57 | 3’-GCTGGCACGCAATCCCAATCCCAATCCCAATCCCAATCCCAATCCCGGTGGAGGAC  G-5’ |

**Table S1: Oligonucleotide Sequences**
